# Integrating fire predisposition assessment into decision support systems for mountain forest management

**DOI:** 10.1016/j.mex.2025.103332

**Published:** 2025-04-25

**Authors:** S. Mutterer, J. Schweier, L.G. Bont, G.B. Pezzatti, M. Conedera, C. Temperli, V.C. Griess, C. Blattert

**Affiliations:** a Sustainable Forestry, Swiss Federal Institute for Forest, Snow and Landscape Research WSL, Birmensdorf, Switzerland; b Forest Resources Management, Department of Environmental Systems Science, ETH Zurich, Zurich, Switzerland; c Insubric Ecosystems, Swiss Federal Institute for Forest, Snow and Landscape Research WSL, Cadenazzo, Switzerland; d Scientific Service National Forest Inventory, Swiss Federal Institute for Forest, Snow and Landscape Research WSL, Birmensdorf, Switzerland

**Keywords:** Decision support system, Disturbance predisposition assessment, Forest fire, Long-term forest planning, Forest disturbances, Multi-criteria decision analysis, Fire predisposition assessment system

## Abstract

Strategic long-term planning of mountain forests in the European Alps requires a balancing act between sustaining forest biodiversity and ecosystem services (BES) and mitigating disturbance risks, particularly under climate change. Multi-criteria decision support systems (DSSs) address this challenge by integrating climate-sensitive forest modelling into frameworks for the evaluation of BES provision under simulated climate and management trajectories. Recent developments incorporate assessments of disturbance predisposition into DSSs, accounting for risks from bark beetle infestations and windthrow. These DSS frameworks have proven flexible applicability across various forest models, spatial scales, forest types, and environmental conditions. However, climate-change-induced transitions of disturbance regimes require adaptations of existing DSS frameworks by accounting for emerging disturbance agents, such as forest fires. Here, we introduce the integration of a fire predisposition assessment system (PAS) into a DSS, incorporating factors such as topography, climatic conditions, wildland–urban interface, and stand structural characteristics. Particularly in the context of long-term planning in mountain forests, the expanded DSS could enable the identification of conflicting forest management objectives related to BES provision and disturbance mitigation efforts under climate change, leading to more informed management decisions.

• A predisposition assessment system for assessing multiple predisposing factors of forest fires is presented.
• The fire PAS enables an integrated evaluation with disturbance predisposition to bark beetle infestations and windthrow, as well as BES provision.
• The modular structure of the fire PAS enables adaptation and application to various spatial scales, as well as integration with different forest models.

## Graphical abstract

**Figure:**
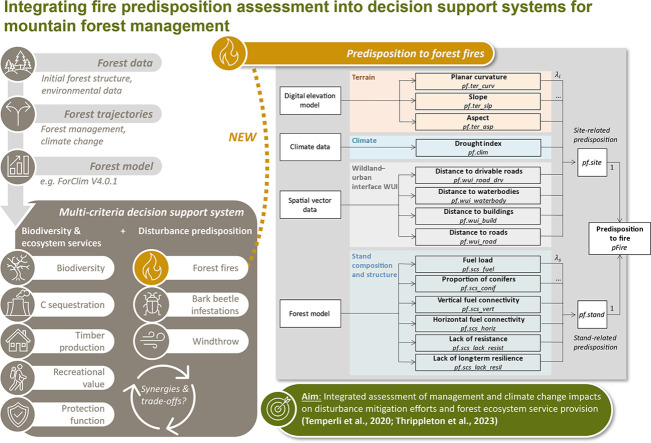

## Specifications table

**Table:** 

Subject area:	Environmental Science
More specific subject area:	Multi-criteria decision support systems for long-term forest planning
Name of your method:	Fire predisposition assessment system
Name and reference of original method:	**Temperli, C.,** Blattert, C., Stadelmann, G., Brändli, U.B., Thürig, E., 2020. Trade-offs between ecosystem service provision and the predisposition to disturbances: a NFI-based scenario analysis. For. Ecosyst. 7 (1), 27. **Thrippleton, T.,** Temperli, C., Krumm, F., Mey, R., Zell, J., Stroheker, S., Gossner, M.M., Bebi, P., Thürig, E., Schweier, J., 2023. Balancing disturbance risk and ecosystem service provisioning in Swiss mountain forests: an increasing challenge under climate change. Reg. Environ. Change 23 (1), 29. **Netherer, S.,** Nopp-Mayr, U., 2005. Predisposition assessment systems (PAS) as supportive tools in forest management—rating of site and stand-related hazards of bark beetle infestation in the High Tatra Mountains as an example for system application and verification. For. Ecol. Manag. 207 (1–2), 99–107.
Resource availability:	The datasets supporting the findings of the related research article are available at 10.5281/zenodo.14627898

## Background

The management of mountain forests in the European Alps is a balancing act between sustaining forest biodiversity and ecosystem services (BES) [1] and mitigating disturbance risks, and it is increasingly challenged by the uncertainties arising under climate change [2]. Decision support systems (DSSs) for strategic long-term forest planning address these challenges by integrating climate-sensitive forest modelling (e.g. by utilizing the forest gap model ForClim [3]) with multi-criteria decision analysis (MCDA) frameworks that consider a broad range of BES indicators [4,5], enabling a comprehensive assessment of different forest management strategies [1,2]. In this way, DSSs aim to support structured decision processes in strategic forest planning by providing a transparent and practical set of decision indicators derivable from forest model outputs [4,5].

To further enhance DSSs focused on BES provision from mountain forests (i.e. biodiversity, carbon sequestration, timber production, recreational value, protection against gravitational hazards; [4,5]), recent developments [2] have incorporated indicator-based assessments of disturbance predisposition to bark beetle infestations and windthrow [6,7]. Such indicator-based assessments of disturbance predisposition [[6], [7], [8]] differ from dynamic simulations of disturbances [9,10] but offer high flexibility for integration into DSS frameworks for BES provision, and they have proven effective across various forest models and spatial scales [2,7]. This approach facilitates the identification of conflicting management objectives, i.e. trade-offs between disturbance mitigation efforts and BES provisioning. Typically, predisposition assessment systems (PASs) are expert-knowledge-based models that enable the evaluation of a broad range of predisposing factors contributing to disturbance risk, including environmental conditions (site-related predisposition) and forest structural characteristics (stand-related predisposition) [6,7]. Individual factors are integrated using an expert-defined weighting system to reflect their relative importance. The resulting predisposition indicators serve as straightforward measures for decision-makers, aiding in the evaluation of management strategies for disturbance mitigation and in the identification of synergies and trade-offs with BES [2].

Given the climate-change-induced transitions of disturbance regimes [11], the existing DSSs for mountain forests require adaptations, such as accounting for risks arising from forest fires [10,12]. While comprehensive frameworks have been developed for the fire risk assessment of current forest conditions [12,13], a module for fire predisposition assessment complementary to the DSS approach, as presented by [2], is rare. To address this gap, we aimed to extend DSS capabilities by integrating a module for a fire PAS. The extended DSS supports informed decision-making in long-term forest planning by addressing a broad range of ecosystem components (through climate-sensitive forest modelling; [1,3]), disturbance agents, and BES.

While this article focuses on the technical integration of the fire PAS into a DSS, a related research article [14] demonstrates its practical application within an expanded DSS. There, the module is implemented within a DSS that holistically evaluates fire, wind and bark beetle predisposition under simulated forest management and climate trajectories, providing information on synergies and trade-offs of disturbance mitigation efforts with BES provision.

## Method details

We present the integration of a fire PAS into the DSS outlined by [2], which currently accounts for site- and stand-related predisposition to bark beetle infestations and windthrow [6,7]. The structure (i.e. the aggregation, assessment, and weighting of individual components) of the fire PAS follows the logic of the predisposition assessment for windthrow and bark beetle infestations [2,6,7]. In this way, the fire PAS maintains methodological consistency within the DSS while offering flexibility for adaptation to different forest models and spatial scales.

### *Linking PAS and DSS*

The integration of a fire PAS into the DSS follows the principles of MCDA [4,15], ensuring the systematic integration of various factors influencing fire predisposition within a standardized and transparent framework. Following MCDA principles, the novel fire PAS involves defining a structure for aggregating (see section ‘A. Aggregation’), assessing (see section ‘B. Assessment’), and weighting individual predisposition components according to their relevance within the PAS (see section ‘C. Weighting’). Further, a sensitivity analysis is performed to assess the robustness of the PAS (see section ‘Method validation’), i.e. an internal validation against variations in component weights and scores [5,12,15]. Following [4,15,16], the criteria for indicator selection were: *relevance* (i.e. the indicator contributes to fire predisposition; indicated by empirical evidence), *practicability* (i.e. the required data can be acquired with reasonable effort), *sensitivity* (i.e. the ability to respond to changes in the assessed system), and the *understandability* of individual indicators to decision-makers.

Within the fire PAS, individual predisposition components were selected to comprise a comprehensive set of factors that influence forest fire predisposition, including indicators related to the sub-groups of terrain [[17], [18], [19]], climatic conditions [9,20], wildland–urban interface (WUI) [12,21,22], and stand structure and composition [10,18,23,24]. In this context, predisposition refers to the degree to which a forest stand (*stand-related predisposition*) and its immediate environment (*site-related predisposition*) are conducive to fire ignition, spread, and extinction, as well as its resistance and resilience to fire events (Table 1), which contribute e.g. to fire frequency and severity.

**Table 1:** Assessment of stand- and site-related fire predisposition, comprising the sub-groups of terrain, climate, wildland–urban interface (WUI), and stand composition and structure. Based on evidence from the scientific literature, the influence of each component on forest fires is indicated as follows: fire ignition (I), fire spread (S), fire extinction (E), and resistance and resilience to fire (R). To express the relative importance of individual components within the overall predisposition score, decision-makers can assign weights to individual components of stand- and site-related predisposition (see section ‘C. Weighting’).

	Sub-group	Component	Influence on fire predisposition	Influence	Refs.	Assessment, data type, and data source
Site-related (*pf.site*)	Terrain	Planar curvature, *pf.ter_curv*	Increasing fire spread with increasing occurrence and degree of canyon-like geomorphometry.	S	[19]	Presence and degree of canyon-like terrain morphology, derived from digital elevation model (DEM), resolution: 2 m, swissALTI3D, swisstopo
		Slope, *pf.ter_slp*	Increasing fire spread with increasing slope.	S	[9,17,18]	Slope at stand centroid, derived from DEM, resolution: 25 m, Shuttle Radar Topography Mission (SRTM)
		Aspect, *pf.ter_asp*	Increasing ignition probability and fire spread with increasing southerly exposition.	S, I	[17,18]	Aspect at stand centroid, derived from DEM, resolution: 25 m, Shuttle Radar Topography Mission (SRTM)
	Climate	Drought index, *pf.clim*	Increasing ignition probability and fire spread with increasing aridity.	S, I	[9,20]	Drought index provided by simulation output from the forest gap model ForClim [3,25]; requires input regarding historical climate and simulated climate trajectories (see e.g. [1].)
	WUI	Distance to buildings, *pf.wui_build*	Increasing ignition probability with decreasing distance to buildings.	I	[12,21]	Euclidean distance of stand centroid to buildings ≥ 75 m^2^ in basal area, Swiss Map Vector 10, swisstopo
		Distance to roads, pf.wui_road	Increasing ignition probability with decreasing distance to roads, paths, hiking trails, etc.	I	[12,17,21]	Euclidean distance of stand centroid to roads and paths, Swiss Map Vector 10, swisstopo
		Distance to drivable roads, *pf.wui_road_drv*	Increasing fire extinction capability with decreasing distance to closest drivable road.	E	[13,22]	Euclidean distance of stand centroid to network of drivable roads (federal and cantonal roads, drivable forest roads with max. bearing capacity ≥ 13 tons), Office for Forest and Natural Hazards, Canton of Grisons
		Distance to waterbodies, *pf.wui_waterbody*	Increasing fire extinction capability with decreasing distance to closest waterbody.	E	[12,13]	Euclidean distance of stand centroid to rivers (status > “very small”, Swiss Map Vector 10, swisstopo) and water extraction points for ground-based and air-based firefighting operations, Office for Forest and Natural Hazards, Canton of Grisons
Stand-related (*pf.stand*)	Stand composition and structure	Fuel load, *pf.scs_fuel*	Increasing fire spread with increasing fuel build-up.	S	[9,18,26]	Stand-level assessment (basal area per ha) based on ForClim simulation output; requires input about initial forest conditions, simulated climate scenarios, and management trajectories (see e.g. [1].)
		Share of conifers, *pf.scs_conif*	Increasing ignition probability and fire spread with increasing share of coniferous species.	S, I	[17,24]	Stand-level assessment (share of coniferous species) based on ForClim simulation output
		Vertical fuel connectivity, *pf.scs_vert*	Increasing fire spread with increasing vertical fuel connectivity.	S	[18,23,24]	Stand-level assessment (coefficient of variation of tree height) based on ForClim simulation output
		Horizontal fuel connectivity, *pf.scs_horiz*	Increasing fire spread with increasing horizontal fuel connectivity.	S	[18,26,27]	Stand-level assessment (stand density index) based on ForClim simulation output
		Lack of resistance, *pf.scs_lack_resist*	Increasing resistance with increasing share of trees resistant to surface fires of moderate intensity.	R	[10,28,29]	Stand-level assessment (post-fire mortality, [10]) based on ForClim simulation output and on allometries for bark thickness [30] and crown length [31]
		Lack of long-term resilience, *pf.scs_lack_resil*	Increasing resilience with increasing share of resilient trees.	R	[12,32]	Stand-level assessment based on ForClim simulation output; classification of species-specific long-term sensitivity to fire [32]

### *DSS context and study site*

The design of the presented fire PAS is closely linked to the related application study in a broader DSS context [14], which is based on forest simulations with ForClim V4.0.1 [3] for a mountain forest enterprise in the Central Alps of Switzerland [1]. Accordingly, the assessment of stand- and site-related predisposition focuses on the level of individual forest stands, representing the smallest spatial unit at which forest management decisions are made [1,2]. In addition, the assessments of the stand-related predisposition were carefully defined to be compatible with the ForClim output (i.e. aligning with the indicator selection criterion of *practicability* [4]). However, the modular character of the fire PAS allows adaptations to other forest models and different spatial scales [2,7].

**Fig. 1:**
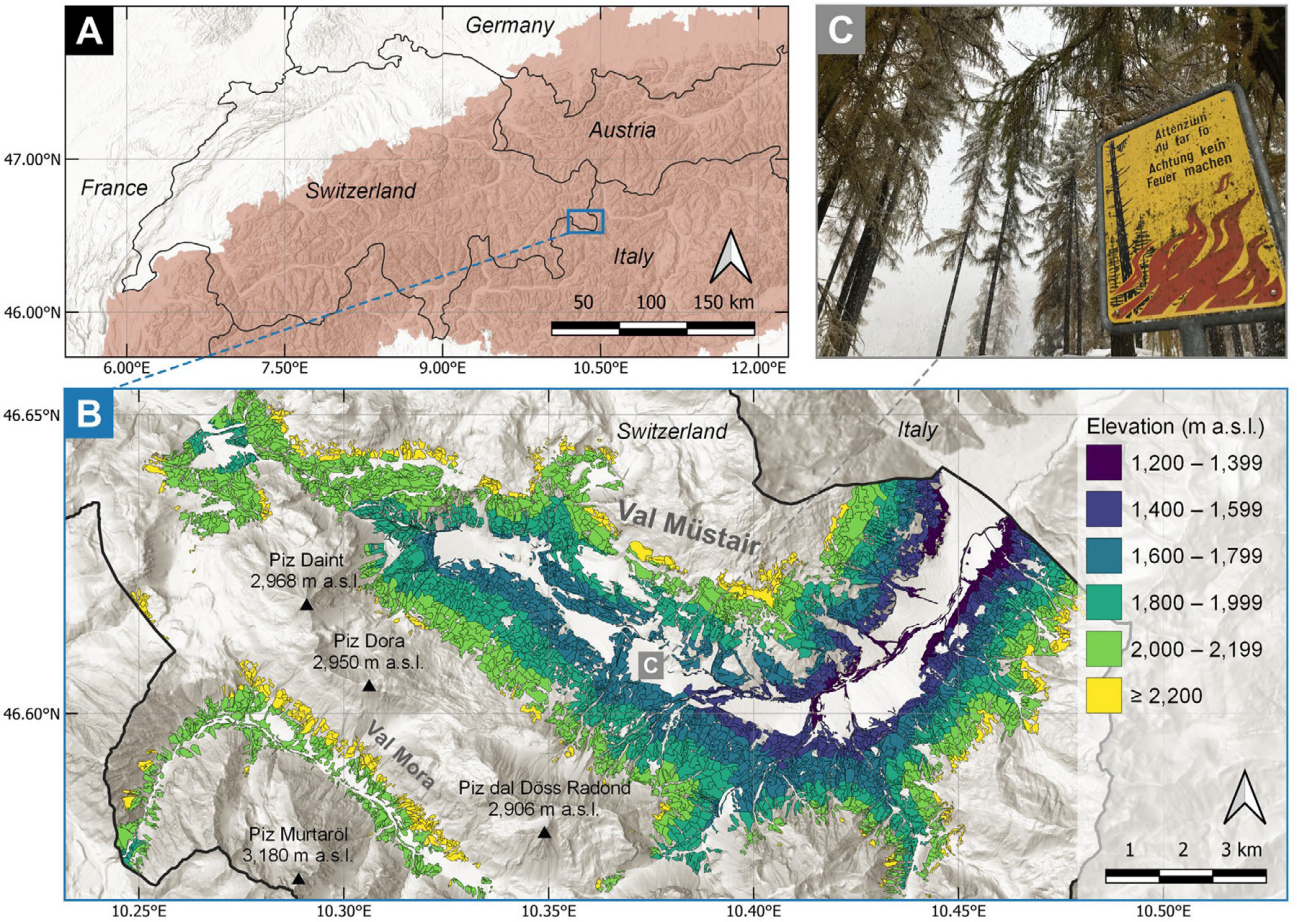
(A) Location of the Val Müstair case study site in Switzerland, (B) distribution of the 5786 forest stands for which site-related predisposition components were assessed, and (C) a sign indicating the prohibition of campfires along a hiking trail to prevent the ignition of forest fires.

To demonstrate the assessment of site-related predisposition and to evaluate the sensitivity of stand-related predisposition components for the simulation of forest trajectories, the Val Müstair forest enterprise was used as a comprehensive case study. Val Müstair is located in the Central Alps of south-eastern Switzerland (46.6°N, 10.4°E). It encompasses a large elevation gradient (ca. 1200–2400 m a.s.l.) and a broad range of topographic conditions. The study area covers 4944 ha of forest, dominated by Norway spruce (*Picea abies*), European larch (*Larix decidua*), and Swiss stone pine (*Pinus cembra*), which is divided into 5786 individual forest stands (Fig. 1). The delineation of the forest stands follows the stand map of the Val Müstair forest enterprise, as provided by the Office for Natural Hazards and Forests (AWN) of the Canton of Grisons. For a detailed description of the site, please refer to the related article [14].

## A. Aggregation: structure of the fire PAS

To aggregate individual predisposition components into an overall predisposition score, the aggregation of the PAS for wind and bark beetles was adopted [2,6,7]. Specifically, equal importance was assigned to stand-related and site-related predisposition, by applying a factor of 0.5 to the weighted sum of each.

The overall disturbance predisposition to fire $pFire_{t,m,c}$ at a given time *t*, under forest management strategy *m* and climate scenario *c*, was calculated as a weighted sum of stand-related and site-related predisposition indicators (Table 1) through an additive function (Eq. (1)).

(1) $$\begin{array}{c} {\mathrm{pFire}}_{t,m,c}=0.5*\sum_{s\in S}\lambda_{s}ST_{s,t,m,c}+0.5*\sum_{i\in I}\lambda_{i}SI_{i,t,m,c} \end{array}$$

where:

**Table:** 

$pFire_{t,m,c}$	Score of disturbance predisposition to fire at time step t, under management strategy m and climate scenario c
S	Set of stand-related predisposition components s, s∈S
I	Set of site-related predisposition components i, i∈I
$ST_{s,t,m,c}$	Predisposition score for each individual stand-related indicator ss∈S at time t, under management strategy m and climate scenario c
$SI_{i,t,m,c}$	Predisposition score for each individual site-related indicator i (i∈I) at time t, under management strategy m and climate scenario c
$\lambda_{s}$	Weight for each stand-related predisposition component s, s∈S, $\sum \lambda_{s}=1$
$\lambda_{i}$	Weight for each site-related predisposition component i, i∈I, $\sum \lambda_{t}=1$
T	Set of time steps, t∈T
M	Set of forest management strategies, m∈M
C	Set of climate scenarios, c∈C

To express the relevance of individual predisposition components in the final additive function, forest fire ecology experts assigned weights to individual components of stand-related ($\lambda_{s}$) and site-related ($\lambda_{i}$) predisposition (see section ‘C. Weighting’). An evaluation of the sensitivity of $pFire_{t,m,c}$ to various weighting scenarios of the individual components (i.e. different values for $\lambda_{s}$ and $\lambda_{i}$), as well as variations in their scores (i.e. different input for $SI_{i,t,m,c}$ and $ST_{s,t,m,c}$) is provided in the ‘Method validation’ section.

## B. Assessment: site-and stand-related predisposition components

The assessment of individual site- and stand-related predisposition components involved two steps:

(1) *Preparation of raw data:* Site-related indicators were calculated based on various types of environmental data (see Table 1 and below), while the computation of stand-related indicators was based on the output of forest simulations under different trajectories of climate change and forest management (i.e. with the forest gap model ForClim, [3]).
(2) *Normalization:* Observed indicator values were assigned a predisposition score between 0 (lowest predisposition) and 1 (highest predisposition) [2,7]. Through this conversion, the raw indicators lost their original units and value ranges, and they were converted into a consistent scale [15], as required for aggregation into the overall predisposition score (Eq. (1)). For indicators not inherently within the 0–1 range, normalization was performed between the 1st percentile (P1) and 99th percentile (P99) of the observed data (e.g. [2].). This P1/P99 normalization made the indicators more robust against outliers and possible extreme values. Depending on the adopted relationship between the raw value and the predisposition, either a linear negative or a linear positive normalization was performed (Table 1). All the lower and upper thresholds used for normalization are provided in Supplementary Table S1.

All analyses were performed in R (version 4.3.1) in the RStudio environment [33].

### B.1. Assessment of site-related predisposition

The assessment of site-related predisposition incorporated indicators of terrain curvature (*pf.ter_curv*), slope (*pf.ter_slp*), aspect (*pf.ter_asp*), and drought (*pf.clim*), as well as distance to roads (*pf.wui_road*), distance to buildings (*pf.wui_build*), distance to drivable roads (*pf.wui_road_drv*), and distance to waterbodies (*pf.wui_waterbody*).

#### B.1.1. Terrain

Terrain influences fire occurrence and behaviour, e.g. by modifying the rate of fire spread along a slope through convection or by affecting site water and energy budgets (e.g. drier and warmer south-facing slopes) [9,17,18]. Two common topographic measures in this context are aspect and slope [17]. Further, on steep slopes, canyon-like terrain morphology might favour eruptive fire behaviour, which is why planar curvature also needs to be considered [19]. A geomorphometric terrain analysis of slope, aspect, and plan curvature was performed with the *MultiscaleDTM* package [34] in R, with window sizes of 175 × 175 m (for slope and aspect) and 18 × 18 m (for planar curvature).

##### Planar curvature (pf.ter_curv)

**Fig. 2:**
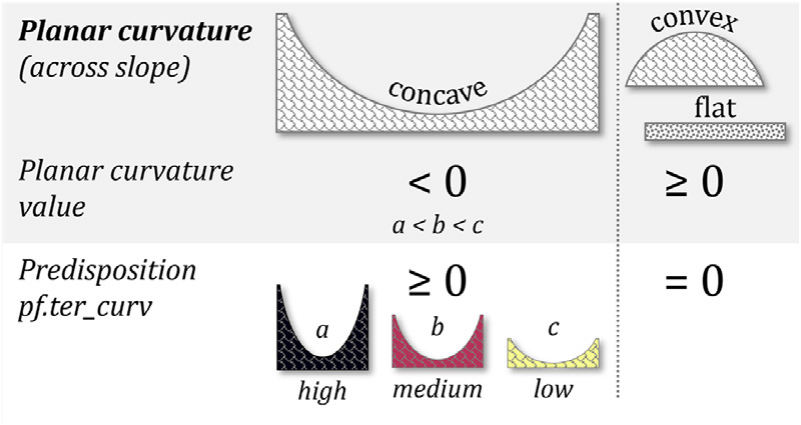
Schematic showing the differentiation of the terrain for the assessment of planar curvature across slope. Planar curvature values < 0, indicating a concave terrain situation (i.e. canyon-like morphometry) were assigned a predisposition score between 0 and 1, depending on the strength of the curvature. A score of *pf.ter_curv* = 0 corresponds to the lowest predisposition, whereas a score of *pf.ter_curv* = 1 corresponds to the highest predisposition. For flat or convex curvature (i.e. ridges), indicated by planar curvature values ≥ 0, a score of *pf.ter_curv* = 0 was assigned.

For *planar curvature*, the presence of canyon-like terrain morphology (indicated by negative curvature values [34]) was analysed based on a high-resolution digital elevation model (DEM; swissALTI3D 2 m, swisstopo). In general, negative curvature values were assigned a predisposition score between 0 and 1, depending on the strength of the (concave) curvature. For flat or convex curvature (i.e. ridges), indicated by curvature values ≥ 0, a predisposition score of 0 was assigned (Fig. 2).

**Fig. 3:**
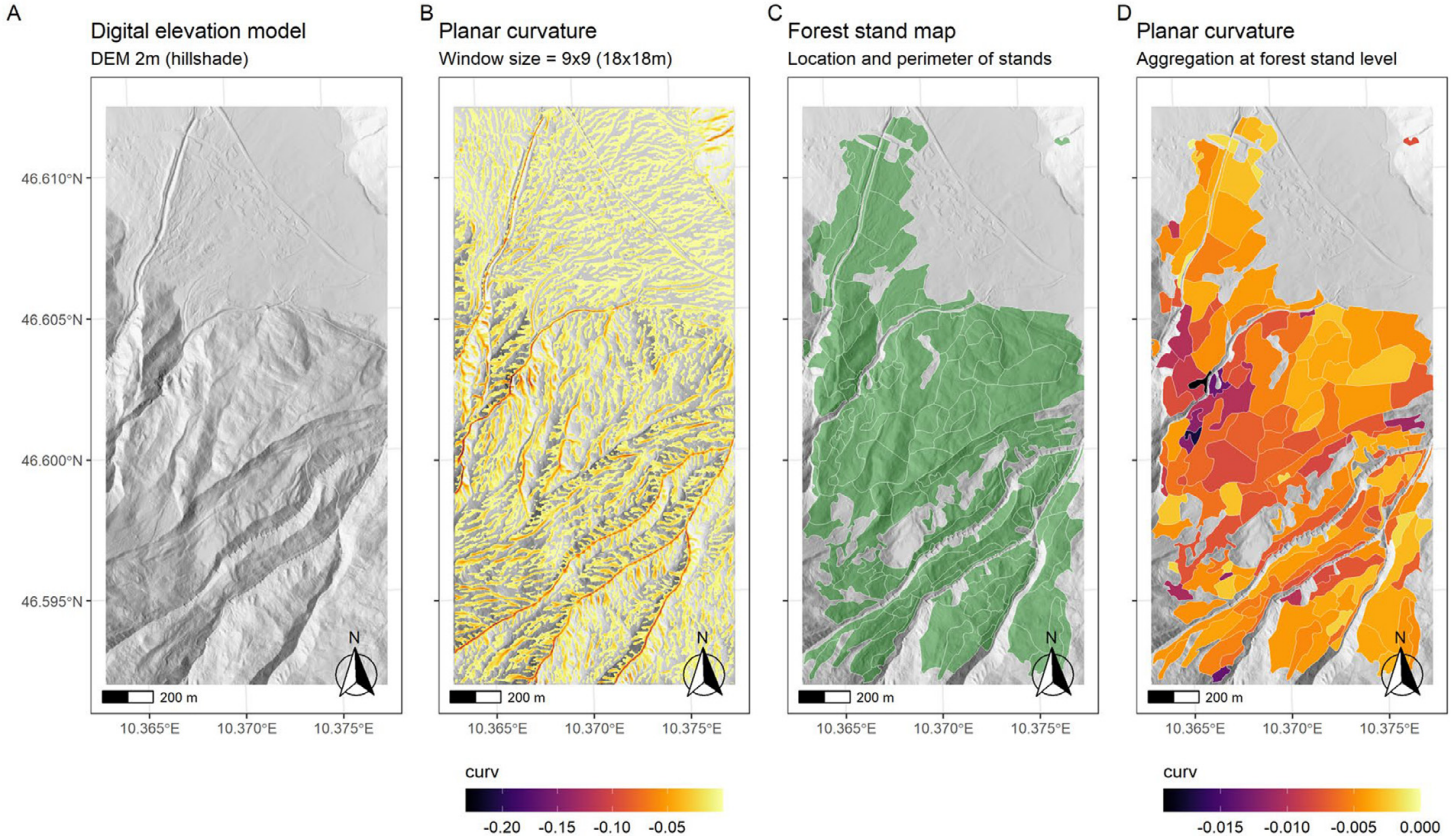
Close-up of a terrain situation in Val Müstair, consisting of: (A) hillshade (based on a digital elevation model [DEM] with 2 m resolution), (B) calculated values for planar curvature, used to identify canyon-like morphometry (only negative values [indicating concave planar curvature] are shown, window size = 9 × 9 raster cells, [34]), (C) locations of forest stands, and (D) aggregation of planar curvature at the level of individual forest stands.

**Fig. 4:**
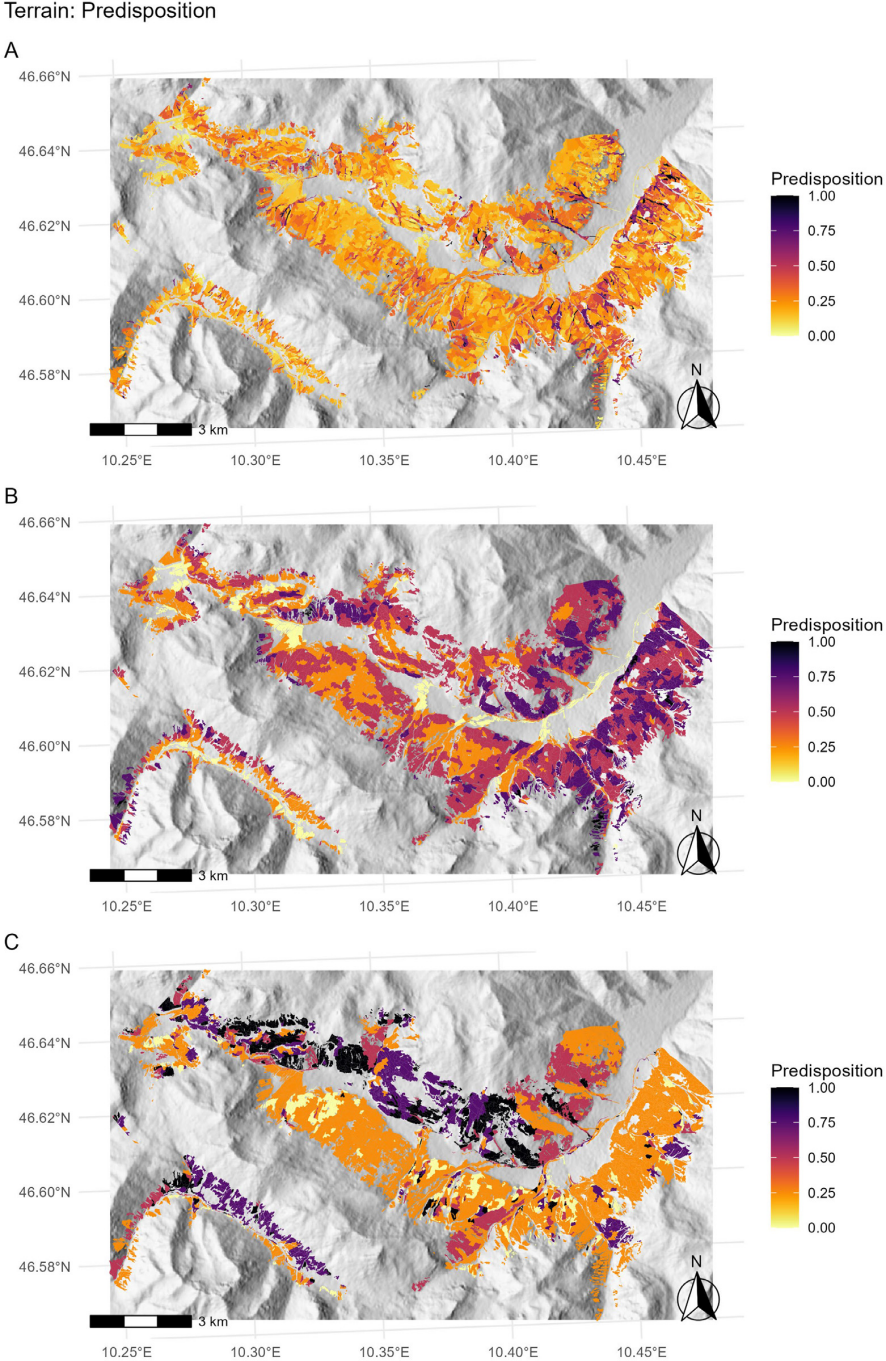
Assigned predisposition scores for (A) planar curvature (*pf.ter_curv*), (B) slope (*pf.ter_slp*), and (C) aspect (*pf.ter_asp*) for all 5786 individual forest stands in Val Müstair.

To aggregate planar curvature at the forest stand level, curvature values > 0 in the 2 × 2 m DEM raster (Fig. 3B) were set to 0, and the mean curvature of the raster cells was then calculated for each stand (Fig. 3C,D, Supplementary Fig. S1A). A linear negative relationship between planar curvature and predisposition was assumed [19]. The highest predisposition (*pf.ter_curv* = 1.00) was assigned to the 1st percentile of observed values for planar curvature, whilst the lowest predisposition (*pf.ter_curv* = 0.00) was assigned to planar curvature values ≥ 0. Thus, for stands comprising only flat or convex terrain, a predisposition score of *pf.ter_curv* = 0 was assigned. In this way, stands with a higher prevalence of negative curvature (i.e. concave) values were considered to have a higher predisposition to forest fire compared with those with a lower prevalence (Fig. 4A). The aggregation reflected not only the share of negative curvature values, but also the degree of the curvature.

##### Slope (pf.ter_slp) and aspect (pf.ter_asp)

Slope and aspect were calculated based on a DEM with 25 m resolution (SRTM) and extracted for centroids of individual forest stands (Supplementary Fig. S1B,C). Predisposition scores for slope and aspect were assigned following the fire danger assessment system for forests in the Austrian Alps (https://www.waldbrand.at, [17]; Fig. 4B,C, Tables 2 and 3).

**Table 2:** Predisposition for slope (*pf.ter_slp*), following the forest fire danger assessment system in the Austrian Alps [17].

Slope	Risk category [17]	Assigned predisposition score *pf.ter_slp*
< 10° and ≥ 50°	very low	0.00
≥ 10 °	low	0.25
≥ 20°	moderate	0.50
≥ 30°	high	0.75
≥ 40°	very high	1.00

**Table 3:** Predisposition for aspect (*pf.ter_asp*), following the forest fire danger assessment system in the Austrian Alps [17].

Aspect class	Risk category [17]	Assigned predisposition score *pf.ter_asp*
N	very low	0.00
W, NW, NE, E	low	0.25
SE	moderate	0.50
SW	high	0.75
S	very high	1.00

#### B.1.2. Climate

Prolonged and intensified drought conditions are among the key drivers of forest fire activity, size, and burn intensity, e.g. by modifying fuel moisture content [10,18,20]. Various fire danger rating systems have been developed, considering the strong influence of climatic variables on fuel conditions and fire behaviour, but also fire occurrence. To evaluate whether and to what extent fire-favouring climatic conditions are becoming more pronounced under climate change, the annual ForClim drought index (FCDI) was applied, as the PAS for bark beetle predisposition already incorporates the FCDI as a sub-indicator for climatic predisposition [7] and it is provided as ForClim simulation output. The FCDI is an adaptation of the *Thornthwaite-Mather* water balance model [35] and incorporates a distinction between soil water supply and demand [25]. Several fire weather indices, such as the Orieux index and the Carrega I87 index, incorporate the water balance model by Thornthwaite and Mather [36] (see also https://wikifire.wsl.ch). The FCDI has been proven to be highly effective in explaining the ignition probability of lightning-induced fires in a southern-Alpine region in Switzerland [20], and it has already been used as proxy for fire spread probability [9].

The annual FCDI refers to months with mean temperatures T_m_ ≥ 5.5 °C and is calculated as:

(2) $$FCDI=1-\sum_{m\in \left(M_{5.5}\right)}\frac{E_{m}}{D_{m}}$$

where $M_{5.5}$ is the set of months with a mean temperature (*T_m_*) ≥ 5.5 °C, *E_m_* is the monthly evapotranspiration, and *D_m_* is the evaporative demand from the soil (for details see [25]).

Alternatively, more established fire weather rating systems [36] could be utilized, depending on the temporal and spatial resolution of the available climatic data (i.e. provided by the corresponding forest model).

**Fig. 5:**
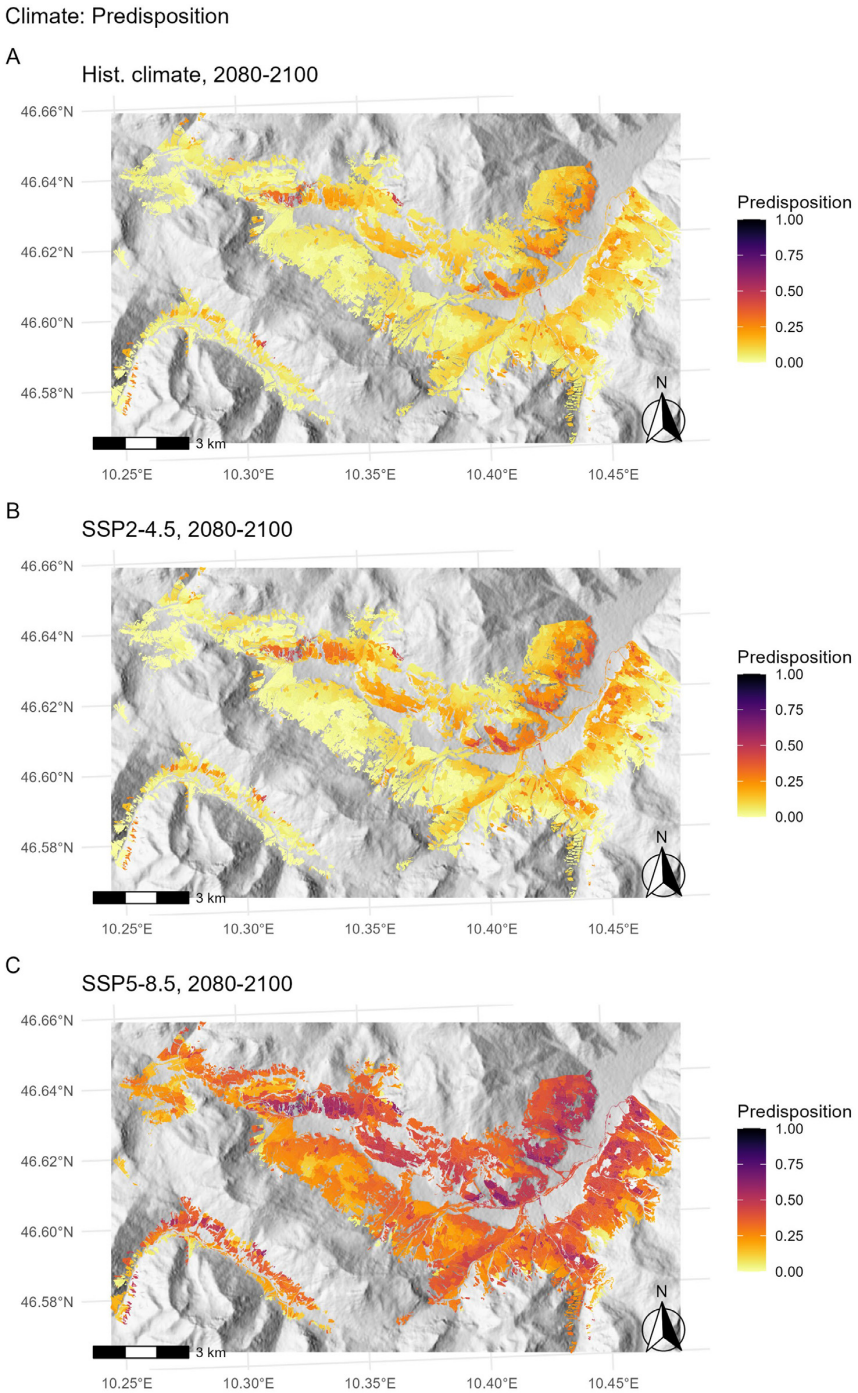
Climatic predisposition (*pf.clim*) for three climate scenarios (A: historical climate, B: climate scenario SSP2–4.5, C: climate scenario SSP5–8.5) based on ForClim V4.0.1 simulation output for the case study site Val Müstair [1]. Predisposition scores are presented as mean values at the stand level over the simulation period 2080–2100.

*Drought index (pf.clim).* Values of FCDI > 0.5 represent extremely dry climatic conditions, which are uncommon in Europe’s forests [3]. To obtain the climatic predisposition score *pf.clim*, a linear positive relationship was assumed between a lower (FCDI = 0) and an upper threshold (FCDI = 0.5), i.e. *pf.clim* was computed by multiplying the FCDI value by two. The FCDI is provided directly by the forest gap model ForClim at the stand level for all simulated time steps and climate trajectories. Here, *pf.clim* was calculated based on the FCDI values simulated in [1], for historical climatic conditions (reference period 1981–2010, Fig. 5A) and for two climate change scenarios (SSP2–4.5 and SSP5–8.5, Fig. 5B,C).

#### B.1.3. Wildland–urban interface (WUI)

The wildland–urban interface (WUI) refers to areas where natural wildland vegetation intersects with anthropogenic infrastructure, including residential areas and roads. In alpine environments, interactions along the WUI are particularly important for the spatial assessment of fire ignition risks [20,21]. For instance, the distance to buildings was found to be an important predictor of the occurrence of human-caused fires in the Swiss cantons of Grisons, Ticino, and Valais [21]. Infrastructure also plays a key role in operational fire management, by ensuring a fast initial response and therefore preventing fires from spreading and reaching critical intensities. This ambiguity can be addressed by considering WUI-related interactions not only as factors that increase the predisposition for anthropogenic ignitions (distance to buildings, distance to roads of all types), but also as factors that foster fire extinction capacities (distance to drivable roads, distance to waterbodies) [12,22].

Euclidean distances from the centroids of individual forest stands to the closest buildings, roads (all categories, including trails and hiking paths), drivable roads, and waterbodies were calculated using the R package *sf* [37].

##### Distance to drivable roads (pf.wui_road_drv) and distance to waterbodies (pf.wui_waterbody)

The Euclidean distances from the centroids of forest stands to drivable roads (Supplementary Fig. S2A) were computed, considering the superordinate road network (i.e. federal and cantonal roads), as well as forest roads with a maximum bearing capacity ≥ 13 t (data provided by the Grisons AWN).

For the distances from the centroids of forest stands to waterbodies (Supplementary Fig. S2B), water extraction points for ground-based and air-based firefighting operations were considered, as documented by the Grisons AWN. Rivers were also considered, utilizing Swiss Map Vector 10, a Swiss national 1:10,000 scale map in vector format that provides detailed spatial information on e.g. road networks, waterbodies, and settlements (Swiss Federal Office of Topography, swisstopo).

**Fig. 6:**
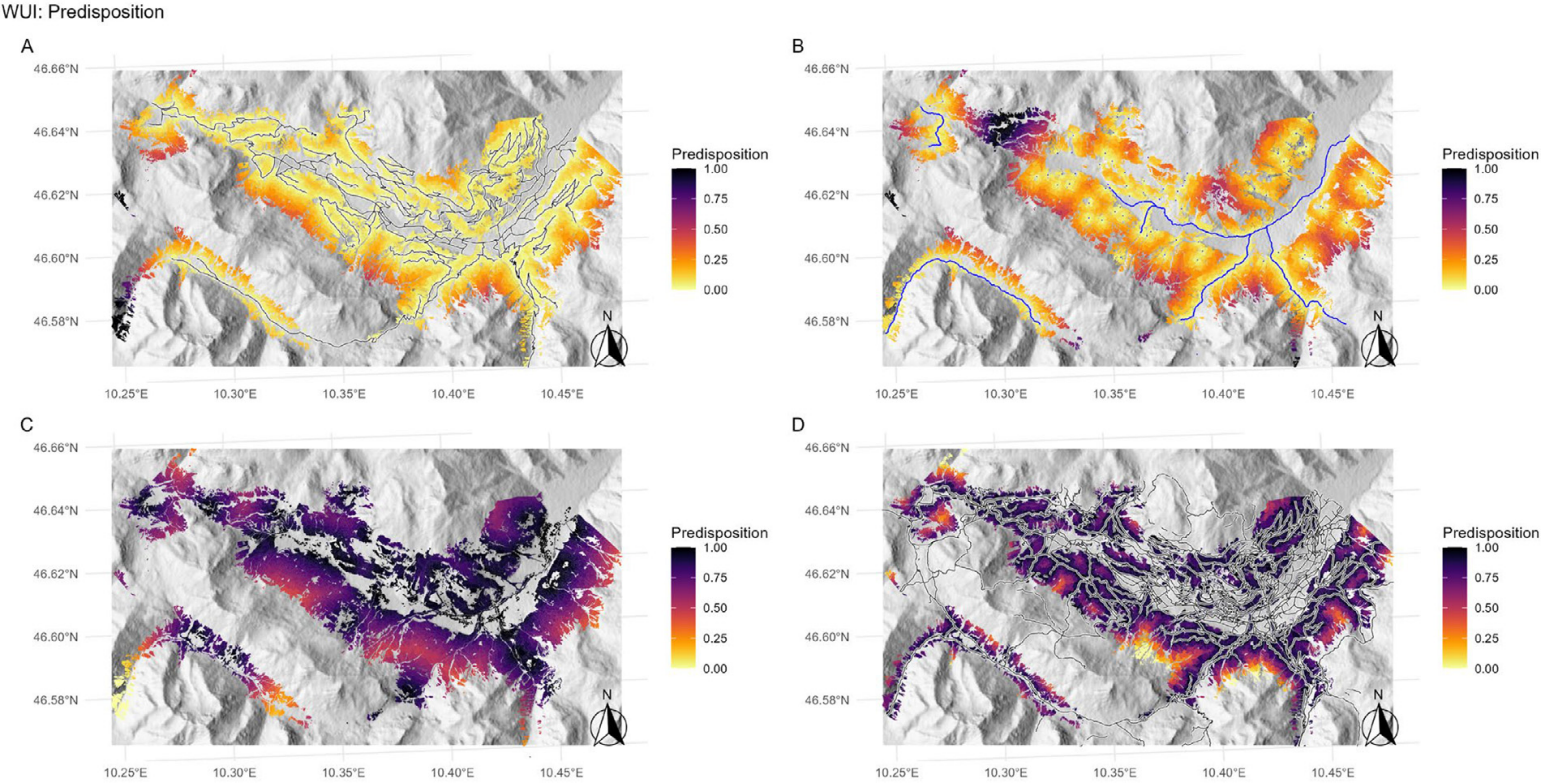
Assigned predisposition scores arising from the wildland–urban interface (WUI). (A) Distances from forest stands to drivable roads (*pf.wui_road_drv*; black lines). (B) Distances from forest stands to the closest waterbody (*pf.wui_waterbody*; rivers: blue lines, water extraction points: blue points). (C) Distances from forest stands to the closest building (*pf.wui_build*; black dots). (D) Distance from forest stands to the closest road, path, etc. (*pf.wui_road*; black lines).

The predisposition components (*pf.wui_road_drv, pf.wui_waterbody*) were derived by assuming a linear positive relationship between calculated distances and predisposition, i.e. values for *pf.wui_road_drv* and *pf.wui_waterbody* were set to 0 at the 1st percentile and to 1 at the 99th percentile of the observed distances, and were linearly interpolated between these points (Fig. 6A,B).

##### Distance to buildings (pf.wui_build) and distance to roads (pf.wui_road)

The calculated distances from the centroids of individual forest stands to buildings and to roads (all types) were based on data retrieved from Swiss Map Vector 10 (Supplementary Fig. S2C,D).

The predisposition components (*pf.wui_build, pf.wui_road*) were derived within the DSS framework by assuming a linear negative relationship between calculated distances and predisposition, i.e. values for *pf.wui_build* and *pf.wui_road* were set to 1 at the 1st percentile and to 0 at the 99th percentile of the observed distances, and were linearly interpolated between these points (Fig. 6C,D).

### B.2. Assessment of stand-related predisposition

*Fuel load (pf.scs_fuel).* The fuel load size is directly related to fire intensity and thus impacts the likelihood of a fire’s transition into a crown fire, also due to its effect on the release of energy from the fire [9,18]. Fuel load can be assessed as the amount of combustible biomass per ha and is frequently further categorized into fuel types, such as surface, shrub, or canopy fuels [9,10,12]. Here, basal area per ha (living trees) was assessed as a simple but effective proxy for fuel-load-related predisposition [26,38], as it can be easily derived from multiple forest simulators. The respective predisposition component *pf.scs_fuel* was derived within the DSS framework by assuming a linear positive relationship between basal area and *pf.scs_fuel*. Specifically, *pf.scs_fuel* was set to 0 at the 1st percentile and to 1 at the 99th percentile of the observed values, and was linearly interpolated between these points.

*Proportion of conifers (pf.scs_conif).* Tree species composition determines various parameters, such as fire spread, extent, and intensity [10,24]. Simplified, pure coniferous forests are perceived as more fire-prone (e.g. due to higher foliage flammability [24]), while mixed forests are considered at intermediate fire risk, and pure deciduous broadleaved forests at low fire risk [17,24]. Consequently, for *pf.scs_conif*, a linear increase in predisposition with increasing conifer share was assumed (*pf.scs_conif* = 0 for 0% conifer share; *pf.scs_conif* = 1 for 100% conifer share).

*Vertical fuel connectivity (pf.scs_vert).* Vertical fuel connectivity, interpreted as a proxy for the availability of so-called ‘fuel ladders’, may increase the risk of a transition from low-intensity surface fires to stand-replacing crown fires [18,24]. Thus, stands with low structural diversity, e.g. measured by the coefficient of variation of tree diameter, can be perceived as less vulnerable, due to the lack of vertical fuel connectivity [23]. Here, the coefficient of variation of tree height was assessed as a proxy for vertical fuel connectivity, expressed as:

(3) $$\begin{array}{c} CV_{h}=\frac{sd_{h}}{\bar{h}} \end{array}$$

with *sd_h_* being the standard deviation and $\bar{h}$ the arithmetic mean of tree height. The respective predisposition component *pf.scs_vert* was derived by assuming a linear positive relationship between *CV_h_* and *pf.scs_vert*. Following this, *pf.scs_vert* was set to 0 at the 1st percentile and to 1 at the 99th percentile of the observed values for *CV_h_*, and was linearly interpolated between these points.

*Horizontal fuel connectivity (pf.scs_horiz).* Active crown fire also requires horizontal fuel connectivity [18], and the stand density index (SDI) was therefore used as a proxy for this connectivity [26,27]. SDI was calculated as:

(4) $$\begin{array}{c} SDI=N*{\left(\frac{dg}{25}\right)}^{1.605} \end{array}$$

with *N* being the number of trees per ha and *dg* the quadratic mean diameter in cm. The respective predisposition component *pf.scs_horiz* was derived by assuming a linear positive relationship between SDI and *pf.scs_horiz*. Following this, *pf.scs_horiz* was set to 0 at the 1st percentile and to 1 at the 99th percentile of the observed values for SDI, and was linearly interpolated between these points.

*Lack of resistance (pf.scs_lack_resist).* Depending on their intensity, wildfires potentially affect a variety of physiological processes in trees [39], which can lead to mortality due to overheated cambial tissue or crown scorch [9,28,40]. To assess the lack of forest resistance to fire (i.e. mortality probability), an established model for post-fire mortality was applied at the level of individual trees [28] and further aggregated to a stand-level estimate [10].

On the individual tree level, the probability of post-fire mortality was modelled as a function of lethal heat-induced damage to the vascular cambium and scorched crown volume. This relationship can be formulated as:

(5) $$\begin{array}{c} P_{m}=C_{k}^{\left(\frac{t_{c}}{t_{f}}-0.5\right)} \end{array}$$

with *P_m_* being the probability of post-fire tree mortality (ranging from 0 to 1), *C_k_* the fraction of scorched crown volume, and *t_c_* the time in seconds required to cause lethal damage to the vascular cambium [28]. The variable *t_f_* constitutes the duration of exposure to fire and was set to 300 s, which is within the typical time range of peak heat exposure during surface fires [10,41].

Following [28]^1^, *t_c_* was estimated as:

(6) $$\begin{array}{c} t_{c}=3.36*bt^{2} \end{array}$$

with *bt* being bark thickness in mm. Bark thickness *bt* was calculated using species-specific allometric relationships between diameter at breast height (*dbh*) and tree height. Allometric relationships are available for several European tree species via the R package *TapeS* [30] (Supplementary Fig. S3A).

Scorched crown volume *C_k_* was calculated as:

(7) $$\begin{array}{c} C_{k}=\frac{\left(h_{k}-h_{t}+cl\right)*\left(h_{t}-h_{k}+cl\right)}{cl^{2}} \end{array}$$

with *h_t_* being tree height (m), *h_k_* height of crown scorch (m), and *cl* crown length (m) [28]. Further, *h_k_* was estimated as:

(8) $$\begin{array}{c} h_{k}=\frac{3.94*FLI^{1.17}}{\left(T_{c}-T_{a}\right)*{\left(0.11*FLI+MFWS^{3}\right)}^{0.5}} \end{array}$$

with *FLI* being fireline intensity (kW/m), *MFWS* mid-flame wind speed (m/s), *T_c_* the lethal temperature threshold of tree tissues (60 °C, [41]), and *T_a_* ambient air temperature (°C) [28]. Given the variety of crown scorch height models [42,43] and the dynamic nature of fire behaviour (i.e. actual fire spread and intensity depend on a broad range of fuel, stand, site and related topography, and weather conditions) [18,19], default values were used here to enable comparisons of stand-inherent resistance under comparable conditions. For the subsequent analysis, *T_a_* (15°), *MFWS* (1.8 m/s), and *FLI* (630 kW/m) were assumed to be constant and were chosen according to values in the literature [18,44]. Supplementary Fig. S4 shows *h_k_* based on various settings for *FLI* and *T_a_*. The default value for *FLI* corresponds to the upper boundary of medium-intensity fires, simulated under dry summer conditions in the French Alps [10]. Consequently, *h_k_* was set to a default value of 19.04 m, representing a rather conservative assumption compared with, e.g. scorch heights of up to 17 m observed by [42].

As information on crown length *cl* is not provided by ForClim, crown allometries were used to calculate the height of the crown base *hcb* as a function of *dbh* and *h_t_*:

(9) $$\begin{array}{c} hcb=h_{t}\left(1-e^{-\left(l_{0}+l_{1}*\left(h_{t}/dbh\right)+l_{2}*dbh\right)}\right) \end{array}$$

with *l_0_, l_1_*, and *l_2_* being species-specific parameters (Table 4, [31]).

**Table 4:** Species-specific coefficients for crown allometries [31] used to calculate crown length.

Tree species / coefficients	*l_0_*	*l_1_*	*l_2_*
*Picea abies*	−0.0443	−0.8823	−0.0004
*Abies alba*	0.1409	−0.8480	−0.0042
*Pinus sylvestris*	0.376	−0.9963	−0.0218
*Fagus sylvatica*	−0.5478	−0.1094	−0.0023
*Quercus petraea*	−0.9967	−0.2043	0.0032

**Fig. 7:**
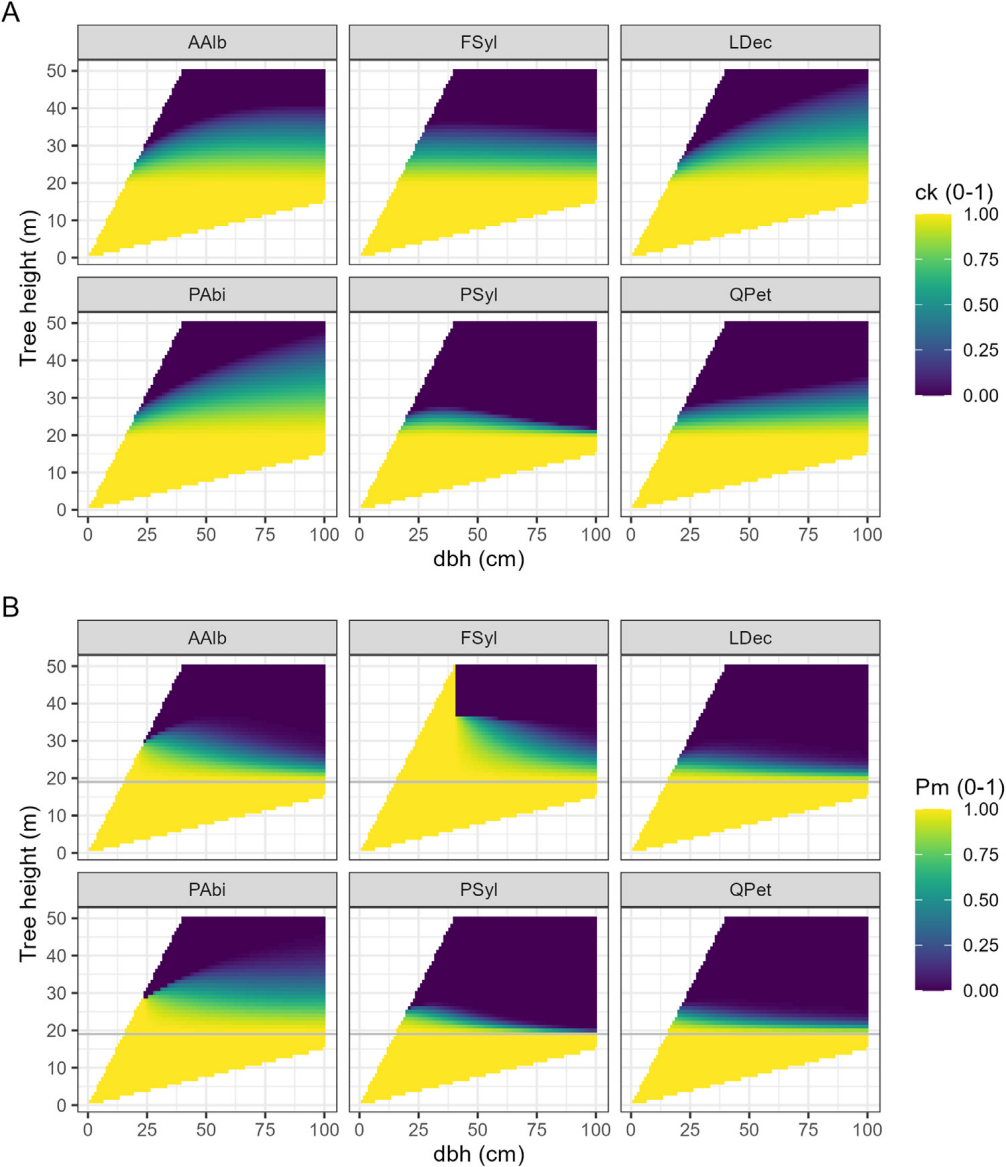
(A) Scorched crown volume (ck) and (B) related probability of post-fire mortality (P_m_) as a function of tree height and diameter at breast height (dbh) for six exemplary tree species (Abies alba, AAlb; Fagus sylvatica, FSyl; Larix decidua, LDec; Picea abies, PAbi; Pinus sylvestris, PSyl; Quercus petraea, QPet). The horizontal grey line in (B) indicates the height of crown scorch (h_k_ = 19.04 m), which was calculated following [28] based on default values for mid-flame wind speed (MFWS; 1.8 m/s) and fireline intensity (FLI; 630 kW/m), according to values from the literature [18,44]. For both (A) and (B), values were calculated for systematic combinations of dbh (in steps of 1 cm) and tree height (in steps of 1 m). The obtained values are only shown for tree height to dbh ratios ranging from 0.15 to 1.25.

Subsequently, crown length was calculated as $cl=h_{t}-hcb$ (Supplementary Fig. S3B). Please note that crown allometry parametrization was only available for *Picea abies, Abies alba, Pinus sylvestris, Fagus sylvatica*, and *Quercus petraea*. Consequently, other species present in ForClim were assigned to one of these species (Supplementary Table S2). Scorched crown volume *C_k_* was estimated as a species-specific function of *dbh* and *h_t_* (Fig. 7A).

In a final step, the probability of post-fire mortality (lack of resistance, *pf.scs_lack_resist*) was calculated at the individual tree level by integrating species-specific sub-models, based on both heat-induced damage to the vascular cambium and scorched crown volume (Eq. (5)). Fig. 7B shows the probability of post-fire mortality (*P_m_*) as a function of *dbh* and *h_t_.* Please note that $P_{m}=1$ for $h_{t}<h_{k}\;\text{and}\;t_{c}<0.5t_{f}$*.*

The estimated values at the individual tree level were then aggregated to a stand-level estimate by calculating the basal-area-weighted mean [10] as:

(10) $$\begin{array}{c} \mathrm{pf}.\mathrm{scs}\_\mathrm{lack}\_\mathrm{resist}\;=P_{m}\left(\mathrm{stand}\right)=\frac{\sum_{i=1}^{n}BA_{i}*P_{m}\left(i\right)}{\sum_{i=1}^{n}BA_{i}} \end{array}$$

with *P_m_(stand)* being the probability of post-fire mortality at the stand level, *BA_i_* being the basal area (in m^2^), and *P_m_(i)* being the probability of post-fire mortality of individual trees *i*.

*Lack of long-term resilience (pf.scs_lack_resil).* Long-term resilience refers to the ability of forests to respond to fires in the long term [12], e.g. through adaptations such as resprouting and fire-induced germination [18]. Here, the lack of long-term resilience was assessed based on a broad review of palaeoecological records of the fire sensitivity of the main tree species in the European Alps [32]. Table 5 provides an overview of the semi-quantitative classification, ranging from extremely resilient to extremely sensitive tree species [32]. Predisposition scores were assigned following this categorization. Values at the individual tree level were aggregated to a stand-level estimate by calculating the basal-area-weighted mean as:

(11) $$\begin{array}{c} pf.scs\_lack\_resil\left(stand\right){=}_{\;}\frac{\sum_{i=1}^{n}BA_{i}*pf.scs\_lack\_resil\left(i\right)}{\sum_{i=1}^{n}BA_{i}} \end{array}$$

with pf.scs_lack_resil(stand) being the predisposition score at the stand level, *BA_i_* being basal area (in m^2^), and pf.scs_lack_resil(i) being the predisposition score of individual trees *i*.

**Table 5:** Semi-quantitative classification of tree species’ long-term sensitivity to fire based on palaeoecological records [32], along with the assigned predisposition score.

Tree species	Sensitivity category (resilience) [32]	Assigned predisposition score *pf.scs_lack_resil(i)*
*Alnus viridis, Castanea sativa, Pinus mugo*	Extremely resilient	0.00
*Alnus* spp., *Corylus avellana, Pinus sylvestris, Quercus* spp., *Salix* spp.	Highly resilient	0.20
*Betula* spp., *Larix decidua*	Resilient	0.40
*Fagus sylvatica, Picea abies, Pinus cembra*	Sensitive	0.60
*Acer* spp., *Fraxinus* spp., *Ostrya carpinifolia, Tilia* spp., *Ulmus* spp.	Highly sensitive	0.80
*Abies alba*	Extremely sensitive	1.00

Alternative assessments can be found e.g. in [12], accounting for tree species’ post-fire regeneration characteristics, such as resprouting and seeding capacity, as well as site-related factors affecting post-fire recovery.

## C. Weighting

To express the relative importance of individual components within the overall predisposition score, decision-makers can assign weights [15,45] to individual components of stand- and site-related predisposition (Eq. (1)). In the related article [14], weights defined by scientists with long-standing expertise in forest fire ecology were applied (Tables 6 and 7).

**Table 6:** Expert-based assessment of weights for the site-related predisposition components $\lambda_{i}$, conducted by combining weights assigned to individual sub-groups $\lambda_{p}$ and to individual components $\omega_{i}$ within each sub-group.

Sub-group (p)	Relative weights of sub-groups ($\lambda_{p}$)	Component (i)	Relative weights of components within sub-groups ($\omega_{i}$)	Final relative weights of components ($\lambda_{i}$)	Normalized weights (${\hat{\lambda }}_{i}$)
Terrain	0.40	*pf.ter_slp*	0.40	0.16	0.53
		*pf.ter_asp*	0.40	0.16	0.53
		*pf.ter_curv*	0.20	0.08	0.27
Climate	0.30	*pf.clim*	1	0.30	1
Wildland–urban interface (WUI)	0.30	*pf.wui_build*	0.25	0.075	0.25
		*pf.wui_road*	0.25	0.075	0.25
		*pf.wui_road_drv*	0.25	0.075	0.25
		*pf.wui_waterbody*	0.25	0.075	0.25

**Table 7:** Expert-based assessment of weights for stand-related predisposition components $\lambda_{s}$, conducted by combining weights assigned to sub-groups $\lambda_{q}$ and to individual components $\omega_{s}$ within each sub-group. Please note that for the assignment of weights, a preliminary division of the sub-group ‘stand composition and structure’ was performed for structured expert judgment.

Sub-group (q)	Relative weights of sub-groups ($\lambda_{q}$)	Component (s)	Relative weights of components within sub-groups $(\omega_{s}$)	Final relative weights of components ($\lambda_{s}$)	Normalized weights (${\hat{\lambda }}_{s}$)
Stand composition and structure: fuel	0.50	*pf.scs_fuel*	0.40	0.20	0.80
		*pf.scs_conif*	0.20	0.10	0.40
		*pf.scs_vert*	0.20	0.10	0.40
		*pf.scs_horiz*	0.20	0.10	0.40
Stand composition and structure: resistance and resilience	0.50	*pf.scs_lack_resist*	0.50	0.25	1
		*pf.scs_lack_resil*	0.50	0.25	1

The definition of the corresponding weighting scenario *expert_weights* involved four steps:

(1) Weights were defined between sub-groups ($\lambda_{p}$ and $\lambda_{q}$), with p being the set of site-related sub-groups (p∈P) and q being the set of stand-related sub-groups, (q∈Q). Each weight ($\lambda_{p}$ and $\lambda_{q}$) was obtained by pairwise comparison of the sub-groups, representing the sub-groups’ relative importance for predisposition compared with the other criteria. The sum of all the weights equals 1 (Eq. (12)).
  (12) $$\begin{array}{c} \sum_{p\in P}^{\;}\lambda_{p}=1\;and\;\sum_{q\in Q}^{\;}\lambda_{q}=1 \end{array}\;$$
(2) Within the sub-groups, local weights were then assigned to individual site-related ($\omega_{i}$) and stand-related ($\omega_{s}$) predisposition components, again by pairwise comparison, representing the components relative importance for predisposition (Eq. (13)).
  (13) $$\begin{array}{c} \sum_{i\in I_{\;}}^{\;}\omega_{i}=1\;and\;\sum_{s\in S_{\;}}^{\;}\omega_{s}=1 \end{array}$$
(3) The final relative weights between individual components across all sub-groups (i.e. final weights $\lambda_{i}$ and $\lambda_{s}$ used in Eq. (1)) were calculated by multiplying the weights from steps 1 and 2 (Eq. (14)).
  (14) $$\begin{array}{c} \lambda_{i}=\lambda_{p}*\omega_{i}\;and\;\lambda_{s}=\lambda_{q}*\omega_{s} \end{array}$$
(4) Further, to improve interpretability (e.g. as presented in [14]), the final relative weights of the individual components were rescaled according to the largest weight by dividing each weight by the maximum final weight (Eq. (15)).
  (15) $$\begin{array}{c} {\hat{\lambda }}_{i}=\frac{\lambda_{i}}{\max\left(\lambda_{i}\right)}\;and\;{\hat{\lambda }}_{s}=\frac{\lambda_{s}}{\max\left(\lambda_{s}\right)} \end{array}\;$$

## Method validation

Internal validation is a key step in MCDA, to assess the robustness and sensitivity of the framework to variations, such as those arising from variations in input and weights [15,46]. By modifying input values, the sensitivity of a PAS to system changes can be evaluated, determining whether it effectively captures relevant responses [46]. In addition, assessing correlations between individual components contributes to the PAS’s interpretability, providing decision-makers with a clearer understanding of how individual framework components interact (e.g. [12]). Here, the validation of the fire PAS involved three steps: (1) assessment of Spearman correlation coefficients between individual components of the indicator framework, (2) evaluation of the sensitivity of the stand-related predisposition (*pf.stand*) to simulated forest management strategies and climate trajectories, to assess its suitability for application in the DSS, and (3) assessment of the impact of different weighting scenarios on the final predisposition score *pFire*. These steps represent a common type of validation for DSS [5,47], as well as for disturbance risk frameworks [12].

To assess the sensitivity of the scores of predisposition components to changes in input (i.e. how they respond to changes in the assessed system), a comprehensive set of ForClim V4.0.1 simulation data were used, covering a broad range of simulated forest development trajectories in Val Müstair until the year 2100 (see [1] and related article [14]) and comprising three climate scenarios (historical climatic conditions, SSP2–4.5, SSP5–8.5). The simulations included six silvicultural management strategies: no management (NO), mountain forest plentering (following the principles of close-to-nature forestry, CNF), CNF with reduced harvesting intensity (CNF-LOW), CNF with increased harvesting intensity (CNF-HIGH), climate-adapted CNF (CNF-ClimAdapt; fostering climate-adapted tree species, following recommendations from tree-app.ch), and high-intensity clearcut management (Clearcut). Clearcut management is not a viable alternative in Swiss mountain forests, as it is prohibited by law, but was simulated to cover a broad spectrum of management strategies applied internationally. Thus, the broad range of simulated forest management and climate trajectories provided a solid basis for testing the indicators’ sensitivity and applicability.

### *Spearman correlation between individual components*

**Fig. 8:**
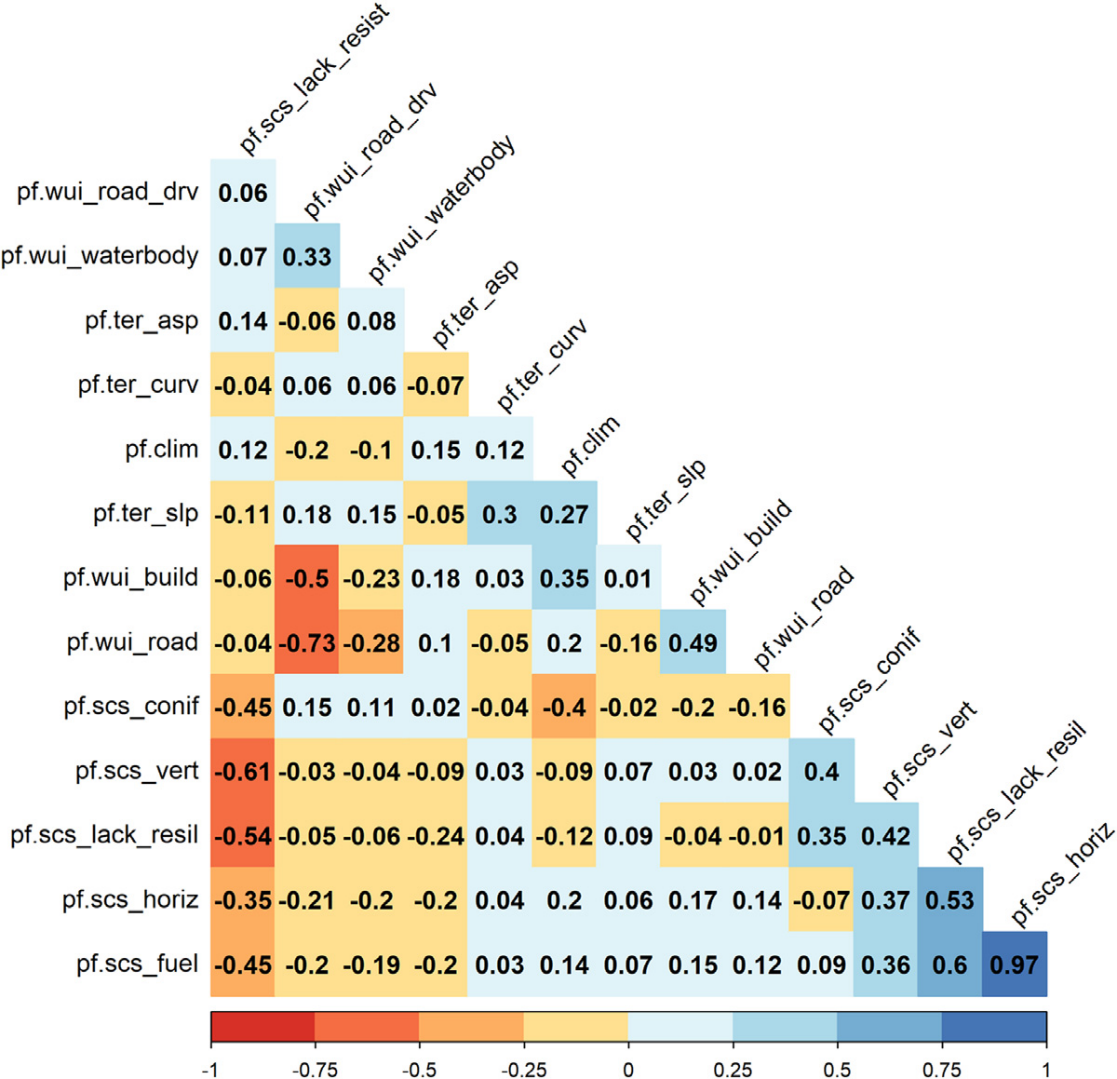
Spearman’s correlations between individual indicators. Correlations were calculated between all simulated forest management strategies and all climate scenarios based on forest growth simulations from [1]. Individual predisposition indicators comprise fuel load (*pf.scs_fuel*), horizontal fuel connectivity (*pf.scs_horiz*), lack of long-term resilience (*pf.scs_lack_resil*), vertical fuel connectivity (*pf.scs_vert*), distance to buildings (*pf.wui_build*), share of conifers (*pf.scs_conif*), distance to roads (*pf.wui_road*), slope (*pf.ter_slp*), planar curvature (*pf.ter_curv*), drought index (*pf.clim*), aspect (*pf.ter_asp*), distance to waterbodies (*pf.wui_waterbody*), distance to drivable forest roads (*pf.wui_road_drv*), and lack of resistance (*pf.scs_lack_resist*).

Spearman correlation coefficients between individual components of the fire PAS were low to moderate in most cases (Fig. 8). Individual moderate to high correlations, particularly between individual stand-related indicators, are causally explainable in that they reflect general relationships between stand indicators simulated with ForClim [1]. The highest positive correlation (r = +0.97) was observed between *pf.scs_horiz* (based on stand density index, SDI) and *pf.scs_fuel* (based on basal area per ha). A noteworthy moderate negative correlation (r = −0.40) was observed between *pf.clim* and *pf.scs_conif*, reflecting the simulated decrease in conifer presence with increasing climate change intensity [1]. Additionally, a strong negative correlation (*r* = −0.73) was observed between *pf.wui_build* and *pf.wui_road_drv*, suggesting the spatial co-occurrence of anthropogenic infrastructure, i.e. buildings and drivable forest roads.

### *Sensitivity of stand-related predisposition components to simulated trajectories*

**Fig. 9:**
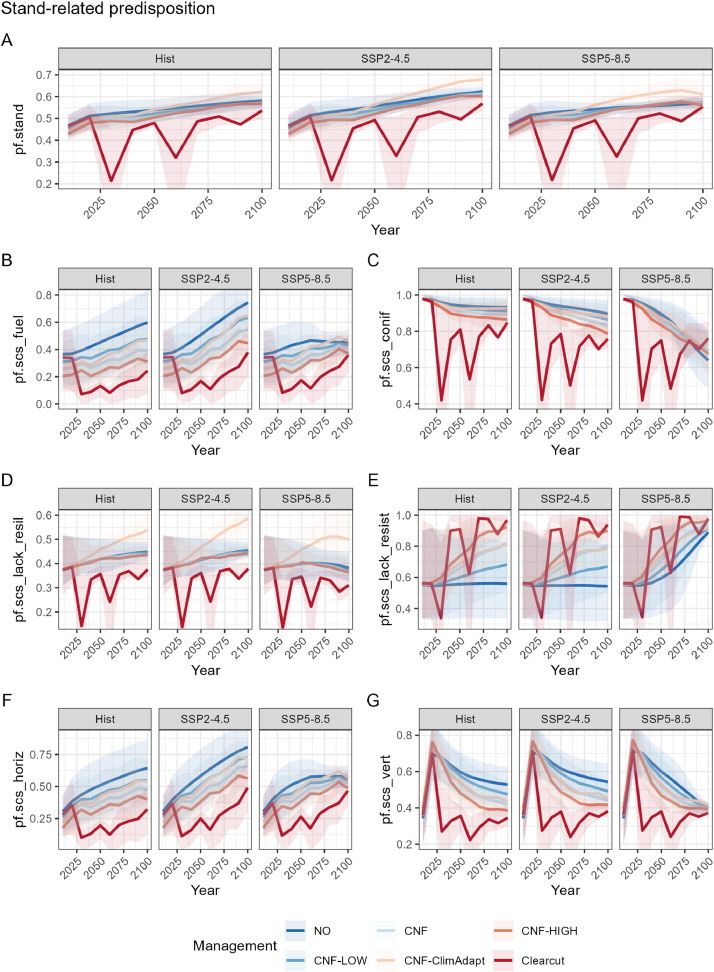
Development of stand-related predisposition under six silvicultural management strategies and three climate scenarios (historical, SSP2–4.5, SSP5.8.5). The six management strategies comprise: no management (NO), mountain forest plentering (following the principles of close-to-nature forestry, CNF), CNF with reduced harvesting intensity (CNF-LOW), CNF with increased harvesting intensity (CNF-HIGH), climate-adapted CNF (CNF-ClimAdapt, fostering of climate-adapted tree species), and high-intensity clearcut management (Clearcut). The mean values over all 5786 simulated forest stands are shown. (A) Aggregated indicator based on weighting scenario ‘*expert_weights*’, (B) fuel load (*pf.scs_fuel*), (C) conifer share (*pf.scs_conif*), (D) lack of resilience (*pf.scs_lack_resil*), (E) lack of resistance (*pf.lack_resist*), (F) horizontal fuel connectivity (*pf.scs_horiz*), and (G) vertical fuel connectivity (*pf.scs_vert*).

The individual indicators used for assessing stand-related predisposition showed sensitivity to the simulated forest management and climate trajectories (Fig. 9; see also the related article [14] for a comprehensive analysis). The observed temporal trends, management intensities, and climate change impacts aligned with observations from a previous study and can be causally explained by simulated forest dynamics [1].

The aggregated stand-related predisposition score (*pf.stand*) under Clearcut management was generally lower and fluctuated more than under the various CNF management variants (Fig. 9A), as a result of the high management intensity in the Clearcut scenario.

The temporal development of the predisposition component *pf.scs_fuel* (Fig. 9B) reflects a fuel build-up with decreasing management intensity, with additional fuel accumulation in the SSP2–4.5 scenario due to the improved forest growth conditions under moderate climate change. A distinct reduction in the proportion of conifers (*pf.scs_conif*) was observed under SSP5–8.5 (Fig. 9C), likely due to unfavourable conditions for conifers under this severe climate scenario [1].

An increasing lack of long-term resilience (*pf.scs_lack_resil*) was observed under the management strategy CNF-ClimAdapt (Fig. 9D). This may be largely attributed to the simulated planting of *Abies alba* [1], a tree species with high adaptation potential to climate change in the case study area of Val Müstair, but which exhibits high sensitivity to fire [32]. Thus, the development of *pf.scs_lack_resil* under CNF-ClimAdapt reveals a general shortcoming in the tree species selection, which took climate adaptation potential into account but not sensitivity to disturbances.

With increasing management intensity, lack of resistance (*pf.scs_lack_resist*) tended to increase (Fig. 9E), as a high management intensity promotes regeneration, which may be characterized by low direct resistance to fire due to the thin bark and small height of young trees [10,28]. Further, a decrease in management intensity (Fig. 9F) led to an increase in horizontal fuel connectivity (*pf.scs_horiz*), which was based on SDI.

A sharp peak in vertical fuel connectivity (*pf.scs_vert*) was observed during the first decades across all management strategies (Fig. 9G), mainly attributed to an initialization effect of the forest simulations. Simulations were initialized with data from the cantonal forest inventory and lacked information on regeneration (due to a measuring threshold of *dbh* ≥ 12 cm). Consequently, ForClim filled the regeneration gap left by the inventory data in the first years of simulation by including the establishment of young trees. An effect of management intensity on *pf.scs_vert* was evident, with higher intensities reducing vertical fuel connectivity.

### *Impact of weighting scenarios on pFire*

The sensitivity of *pFire* to the weighting of individual components was evaluated using four weighting scenarios (Table 8): equal weighting of all components (*eq_weights*), equal weighting at the sub-group level (*eq_weights_group*), expert-defined weights (*expert_weights,* see section ‘C. Weighting’), and randomly assigned weights (*random_weights*).

**Table 8:** Four weighting scenarios were defined to evaluate the sensitivity of the overall predisposition score to shifts in indicator weights. Weighting scenarios: equal weighting of all components (*eq_weights*), equal weighting at the level of sub-groups (*eq_weights_group*), expert-defined weights (*expert_weights*, see section ‘C. Weighting’), and randomly assigned weights (*random_weights*). The indicated weights reflect the ratios between the components, i.e. for the calculation of pFire (Eq. (1)), it was ensured that the weights of both the site- and the stand-related components summed to 1, by dividing the weight of each individual component by the sum of all site- and stand-related weights, respectively.

	Sub-group	Component	*eq_weights*	*eq_weights_group*	*expert_weights*	*random_weights*
Site-related (*pf.site*)	Terrain	planar curvature, *pf.ter_curv*	1	0.33	0.53	0.19
		slope, *pf.ter_slp*	1	0.33	0.53	0.83
		aspect, *pf.ter_asp*	1	0.33	0.27	0.32
	Climate	drought index, *pf.clim*	1	1	1	0.36
	Wildland–urban interface (WUI)	distance to buildings, *pf.wui_build*	1	0.25	0.25	0.89
		distance to roads, *pf.wui_road*	1	0.25	0.25	0.21
		distance to drivable roads, *pf.wui_road_drv*	1	0.25	0.25	0.19
		distance to waterbodies, *pf.wui_waterbody*	1	0.25	0.25	0.39
Stand-related (*pf.stand*)	Stand composition and structure	fuel load, *pf.scs_fuel*	1	0.17	0.80	0.16
		share of conifers, *pf.scs_conif*	1	0.17	0.40	0.39
		vertical fuel connectivity, *pf.scs_vert*	1	0.17	0.40	0.62
		horizontal fuel connectivity, *pf.scs_horiz*	1	0.17	0.40	0.71
		lack of resistance, *pf.scs_lack_resist*	1	0.17	1	0.98
		lack of long-term resilience, *pf.scs_lack_resil*	1	0.17	1	0.66

**Fig. 10:**
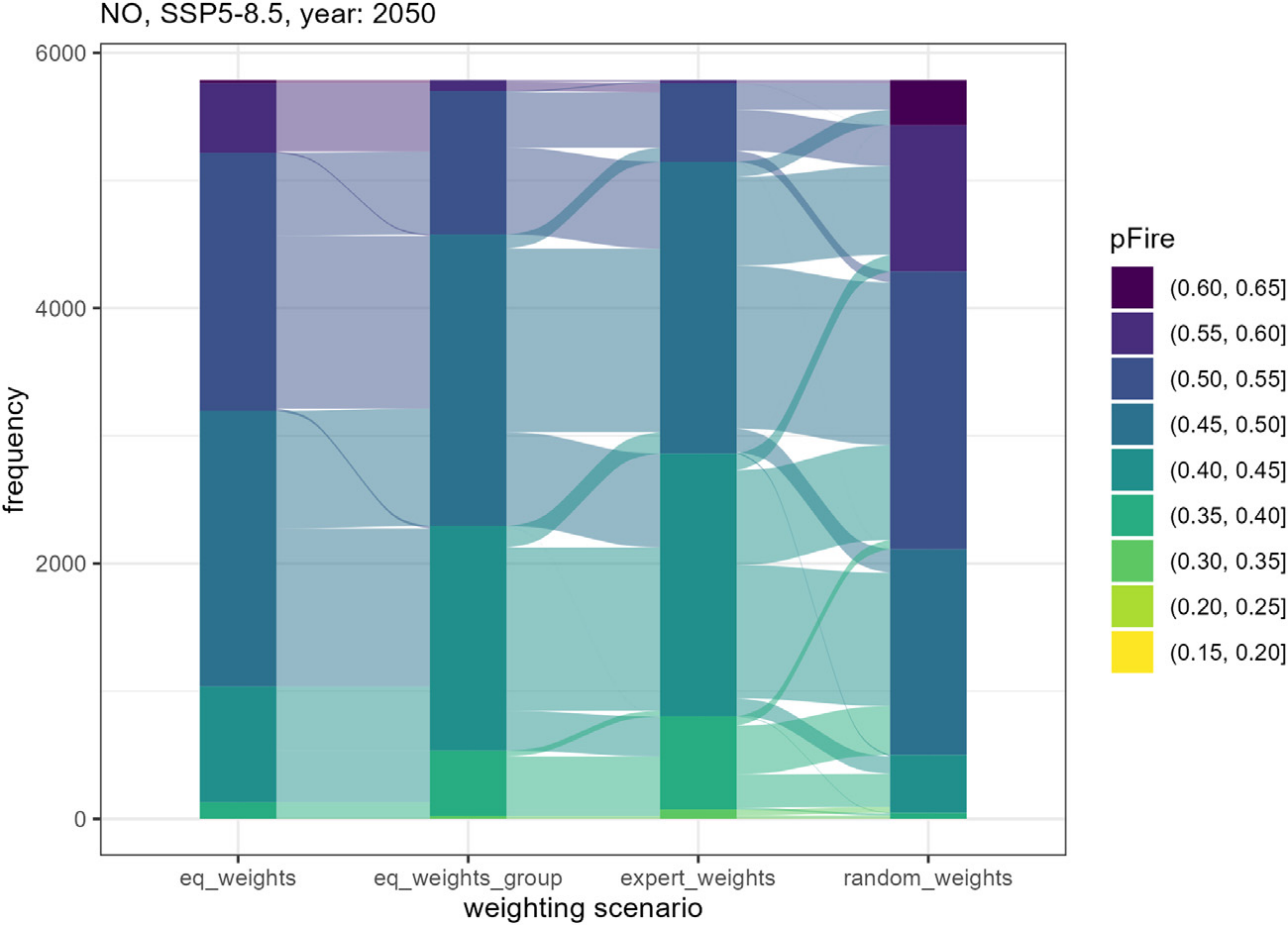
Flow diagram illustrating the distribution of predisposition to fire (*pFire*) scores across different weighting scenarios. Weighting scenarios: equal weighting of all components (*eq_weights*), equal weighting at the level of sub-groups (*eq_weights_group*), expert-defined weights (*expert_weights*), and randomly assigned weights (*random_weights*). pFire was calculated for 5786 forest stands based on the no management strategy (NO) under the climate change scenario SSP5–8.5 in the year 2050. The *pFire* scores are categorized into groups by steps of 0.05. The width of each flow represents the frequency of forest stands within each *pFire* group.

Correlation coefficients were calculated to assess the sensitivity of *pFire* under these four weighting scenarios (as done by e.g. [12]). Spearman correlations were high between the four weighting scenarios (Table 9). The high correlations indicate that all weighting scenarios were capable of capturing the same general trends regarding the development of *pFire* under the simulated climate and management trajectories. Moreover, it can be concluded that the weighting led to shifts in the predisposition scores, but these shifts were moderate (Fig. 10) and did not fundamentally alter the distribution pattern (Supplementary Fig. S5).

**Table 9:** Spearman’s correlation coefficients between predisposition to fire (*pFire*) calculated based on four different weighting scenarios (*n* = 5786 forest stands × 10 simulation periods × 3 climate scenarios × 6 management strategies = 1,041,480 simulated states). Weighting scenarios (see Table 8): equal weighting of all components (*eq_weights*), equal weighting at the level of sub-groups (*eq_weights_group*), expert-defined weights (*expert_weights*, see section ‘C. Weighting’), and randomly assigned weights (*random_weights*).

	*eq_weights*	*eq_weights_group*	*expert_weights*	*random_weights*
*eq_weights*	1	0.967	0.896	0.808
*eq_weights_group*	0.967	1	0.938	0.787
*expert_weights*	0.896	0.938	1	0.860
*random_weights*	0.808	0.787	0.860	1

**Fig. 11:**
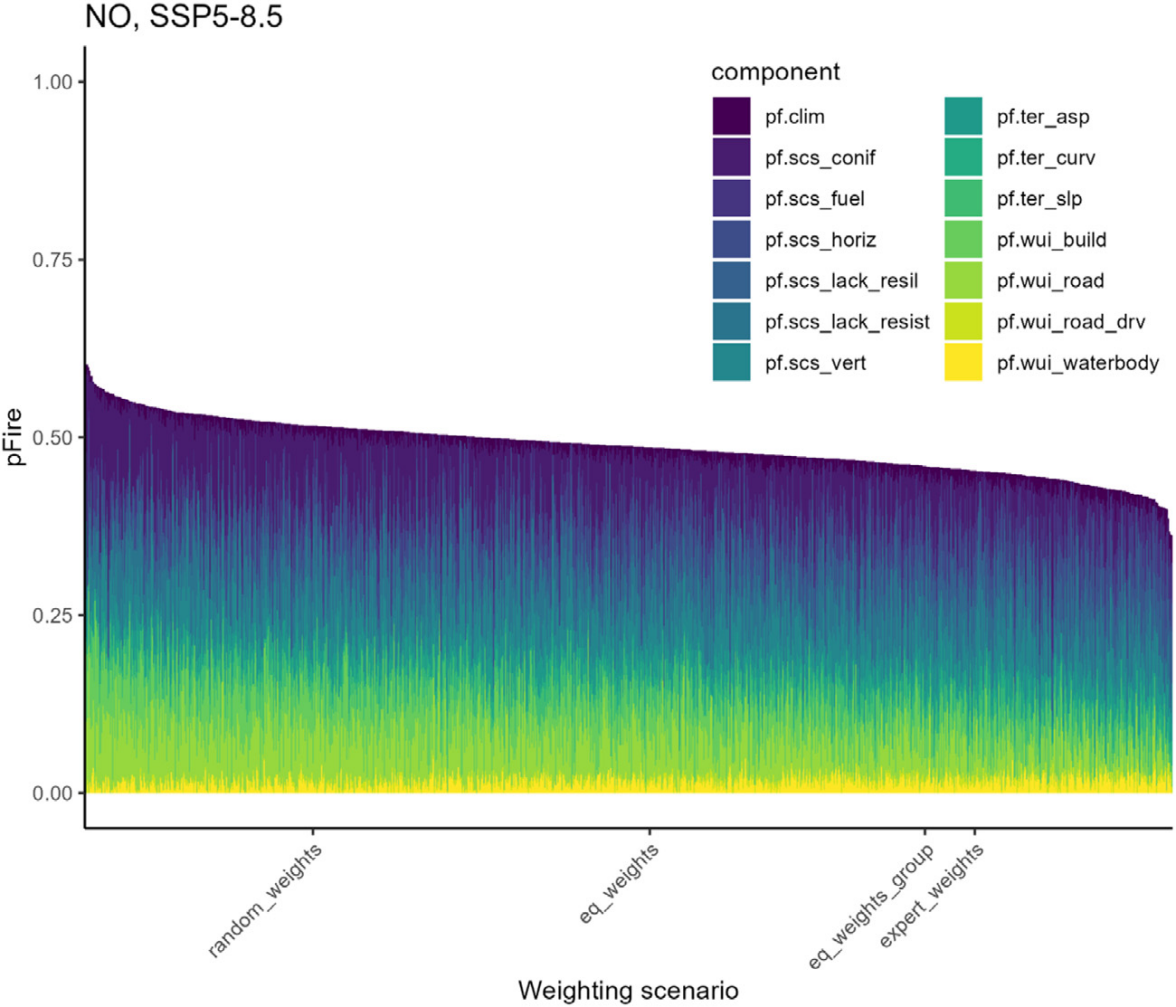
Mean predisposition to fire (*pFire*) for 1000 random weighting scenarios (weights of components randomly assigned to values between 0 and 1, by steps of 0.1) and four predefined weighting scenarios for the no management strategy (NO) and the SSP5–8.5 climate scenario. Predefined weighting scenarios: equal weighting of all components (*eq_weights*), equal weighting at the level of sub-groups (*eq_weights_group*), expert-defined weights (*expert_weights*), and randomly assigned weights (*random_weights*). The respective *pFire* scores are shown in descending order. *pFire* comprises components related to climatic conditions (*pf.clim*), terrain (*pf.ter_slp, pf.ter_asp, pf.ter_curv*), wildland–urban interface (*pf.wui_build, pf.wui_road, pf.wui_road_drv, pf.wui_waterbody*), and stand characteristics (*pf.scs_fuel, pf.scs_conif, pf.scs_lack_resist, pf.scs_lack_resil, pf.scs_horiz, pf.scs_vert*).

In addition, *pFire* was calculated under 1000 random weighting scenarios, where weights for individual components were randomly assigned values between 0 and 1 in steps of 0.1. It was ensured that the weights of both the site- and the stand-related components summed to 1 (see Eq. (1)), by dividing the weight of each individual component by the sum of all site- and stand-related weights, respectively. The distribution of *pFire* under these random weighting scenarios, as well as the positions of the values of *pFire* under the four predefined weighting scenarios, demonstrate that values of *pFire* under the weighting scenario *expert_weights* were within the range of the random weighting scenarios, with a tendency towards the lower end of the range (Fig. 11).

Overall, *pFire* demonstrated sensitivity to changes in the weighting scenario (Figs. 10 and 11). However, these changes were moderate, and the fundamental form of the indicator distribution remained consistent (Supplementary Fig. S5), as already indicated by the high Spearman correlation between *pFire* values under the four predefined weighting scenarios (Table 8). By assigning specific weights, it is possible to adapt *pFire* to case-study-specific requirements (as done here via the *expert_weights* scenario), while at the same time the general trends may be identifiable across several scenarios.

## Limitations

The aim of the approach presented here is to integrate a fire PAS into DSSs for mountain forest management, to address the increasing need to simultaneously account for disturbance mitigation efforts and BES provision in long-term forest planning. At this point, it is important to highlight that the PAS was not designed for the explicit simulation of fire impacts on forests [e.g. 9,10]. While individual components of the fire PAS may be empirically validated (e.g. through comprehensive validations of post-fire mortality models [29], or based on predictor importance for fire occurrence [48]), validating the overall fire predisposition score against external data is not practical due to its integration of diverse concepts (such as fire ignition, fire spread, fire extinction capacities, and species-specific fire adaptation traits), each requiring distinct empirical validation approaches. Instead, the fire PAS is more intended as a method to systematically assess various predisposing factors to forest fires, and the aggregation of the various components ensures operability within the broader DSS application, enabling an integrated assessment with disturbance predisposition to bark beetle infestations and windthrow, as well as BES provision [2,7].

General limitations of the PAS approach can arise from various steps, including the assessment, normalization, weighting, and aggregation of individual predisposition components, corresponding to the limitations of MCDA [15].

(1) Assessment: The structural complexity of the indicators utilized within the presented fire PAS was kept low, to ensure compatibility with the PAS for bark beetle infestations and windthrow [2,7]. For this reason, coupling of the indicators was not implemented. Interconnections between indicators might be relevant, however; for example, the importance of the indicator *pf.scs_vert* for assessing the predisposition to crowning might vary depending on the share of conifers. However, this simplification makes it easier to study the contributions of individual components when interpreting the aggregated predisposition scores.

Further, the fire PAS was tailored for application within a multi-criteria DSS for mountain forests [14], and calculations of stand-related predisposition were based on simulations from the forest gap model ForClim V4.0.1 [3]. To ensure the enforceability of stand-related indicators (i.e. assessments can be conducted with reasonable effort from forest simulation output), simplifications were required. For example, fuel load (*pf.scs_fuel*) was assessed using basal area as a proxy, which may correlate positively with the accumulation of litter and dead woody fuels but may be inversely correlated with live understorey fuel loading [49]. Further, allometric functions were applied to obtain metrics (i.e. crown length and bark thickness) required for the calculation of the indicator *pf.scs_lack_resist*. As allometric functions for crown metrics that are compatible with ForClim output were rare and limited to a few tree species, future efforts should integrate additional species-specific crown allometries as they become available.

(2) Normalization: The normalization applied in the analysis was based on the 1st and 99th percentiles of the observed values at the case study site (for *pf.ter_curv, pf.wui_road_drv, pf.wui_waterbody, pf.wui_build, pf.wui_road, pf.scs_fuel, pf.scs_vert*, and *pf.scs_horiz*), meaning that predisposition needs to be interpreted as a relative rather than an absolute measure [2]. This applies to all normalizations using case-study-specific values, making the PAS case-study specific and reflecting mainly relative differences among the simulated trajectories. Applying the PAS to other case studies would require repeating the normalization for a study site large enough to ensure sufficient data variability. Here, due to the large case study site, a large range of values occurred (Supplementary Table S1). In smaller study areas, however, default threshold values might need to be defined. In this context, it must also be noted that linear relationships were assumed here between the lower and upper thresholds for normalization. For further developments, our framework provides the flexibility to substitute these simplified linear relationships with non-linear normalization approaches, e.g. by accounting for a non-linear relationship between drought indices and fire spread probability [9].

(3) Weighting and aggregation of components: The overall fire predisposition score demonstrated sensitivity to changes in the weighting of individual components. Depending on the intended application of the fire PAS, the weighting might need to be adapted, e.g. to fit with local requirements or different valuations of indicator relevance. Further, the additive function used to aggregate individual predisposition components (Eq. (1)) corresponds to a compensatory MCDA aggregation method [15], i.e. low predisposition scores of a component may be compensated by higher predisposition scores of another component (e.g. as indicated by negative correlations between components, Fig. 8). Thus, we highly recommend providing information on individual predisposition components alongside overall predisposition scores, thereby ensuring the interpretability of observed responses to simulated trajectories.

Further limitations arise from the resolution: The PAS is focused on individual forest stands, a spatial reference level commonly used in DSS studies [1,2], representing the smallest spatial unit at which forest management decisions are made. However, the assessment of site-related predisposition could also be adapted to a gridded approach [17], preventing the loss of high-resolution data due to aggregation at the stand level (as done for e.g. planar curvature, slope, and aspect). Such a gridded analysis would enable a more detailed, fine-grained assessment of site-specific fire predisposition, particularly in relation to terrain morphometry. For stand-related predisposition, interactions between the predispositions of neighbouring forest stands [50] and fuel connectivity along slopes [51] were not considered.

Despite these limitations, the fire PAS presented here offers flexibility regarding adaptation and application to various spatial scales and integration with different forest models, due to its modular structure and flexible data inputs. In future research, this system could be applied to a wide range of biogeographic mountain forest conditions across the European Alps. In addition, the modular structure can handle the integration of additional components, such as those addressing interactions between neighbouring forest stands or a more nuanced assessment of fuel loads.

## Related research article

Mutterer S, Blattert C, Bont L, Griess VC, Schweier J (2025): *Beetles, wind, and fire: effects of climate change and close-to-nature forestry on disturbance predisposition and ecosystem service trade-offs. Forest Ecology and Management, 2025, 586, 122,690.* 10.1016/j.foreco.2025.122690.

## Ethics statements

Not applicable.

## CRediT authorship contribution statement

**S. Mutterer:** Writing – original draft, Conceptualization, Data curation, Investigation, Formal analysis, Methodology, Visualization, Validation. **J. Schweier:** Conceptualization, Writing – review & editing, Supervision, Project administration, Funding acquisition. **L.G. Bont:** Conceptualization, Methodology, Writing – review & editing, Supervision. **G.B. Pezzatti:** Methodology, Writing – review & editing. **M. Conedera:** Methodology, Writing – review & editing. **C. Temperli:** Methodology, Writing – review & editing. **V.C. Griess:** Writing – review & editing. **C. Blattert:** Conceptualization, Methodology, Data curation, Writing – review & editing, Supervision.

## Declaration of competing interest

The authors declare that they have no known competing financial interests or personal relationships that could have appeared to influence the work reported in this paper.

## Appendix Supplementary materials

Image, application 1

## Footnotes

1 Please note: Compared to the formulation in [28], we adjusted units (cm to mm, min to s) and set the parameter “T_1_: assumed instantaneous fire temperature ( °C)” to 200 °C. This customization allows for a straightforward substitution by other models for bark insulation capability, e.g. as presented by [41].
