# Supplementary material for: Integrating fire predisposition assessment into decision support systems for mountain forest management
Source: MethodsX. 2025 Apr 25;14:103332. doi: 10.1016/j.mex.2025.103332 (PMC12090305; doi:10.1016/j.mex.2025.103332)
Supplement: Supplementary file 1 [file mmc1.docx]

Supplementary Material

**Integrating fire predisposition assessment into decision support systems for mountain forest management**

Mutterer, S^a,b^; Schweier, J^a^; Bont, LG^a^; Pezzatti, GB^c^; Conedera, M^c^; Temperli, C^d^; Griess, VC^b^; Blattert, C^a^

^a^ Sustainable Forestry, Swiss Federal Institute for Forest, Snow and Landscape Research WSL, Birmensdorf, Switzerland

^b^ Forest Resources Management, Department of Environmental Systems Science, ETH Zurich, Zurich, Switzerland

^c^ Insubric Ecosystems, Swiss Federal Institute for Forest, Snow and Landscape Research WSL, Cadenazzo, Switzerland

^d^ Scientific Service National Forest Inventory, Swiss Federal Institute for Forest, Snow and Landscape Research WSL, Birmensdorf, Switzerland

**Related research article**

Mutterer S, Blattert C, Bont L, Griess VC, Schweier J (2025): *Beetles, wind, and fire: effects of climate change and close-to-nature forestry on disturbance predisposition and ecosystem service trade-offs. Forest Ecology and Management, 2025, 586, 122690.* [*https://doi.org/10.1016/j.foreco.2025.122690*](https://doi.org/10.1016/j.foreco.2025.122690)

The datasets supporting the findings of the related research article are available at <https://doi.org/10.5281/zenodo.14627898>

*Supplementary Table S1: To obtain predisposition scores for individual components, linear normalization (either linear negative or linear positive) was performed between a lower and upper threshold of observed raw values. For all listed components (except pf.clim), the lower limit was derived from the 1st percentile and the upper limit from the 99th percentile of observed raw values. For pf.clim, which was calculated based on the ForClim drought index FCDI, the lower limit was set to 0 and the upper limit to 0.5.*

| Component | Normalization | Lower | Upper | Unit |
| --- | --- | --- | --- | --- |
| *planar curvature,*  *pf.ter_curv* | linear negative | -0.02260571 | 0.00 | [-] |
| *distance to buildings, pf.wui_build* | linear negative | 59.00 | 2935.00 | [m] |
| *distance to roads,*  *pf.wui_road* | linear negative | 2.43 | 818.97 | [m] |
| *distance to drivable road,*  *pf.wui_road_drv* | linear positive | 6.76 | 2489.92 | [m] |
| *distance to waterbodies,*  *pf.wui_waterbody* | linear positive | 14.00 | 1945.00 | [m] |
| *drought index,*  *pf.clim* | linear positive | 0.00 | 0.50 | [-] |
| *fuel load,*  *pf.scs_fuel* | linear positive | 0.00 | 38.80 | [m^2^ ha^-1^] |
| *horizontal fuel connectivity,*  *pf.scs_horiz* | linear positive | 0.00 | 884.15 | [-] |
| *vertical fuel connectivity,*  *pf.scs_vert* | linear positive | 0.00 | 2.33 | [-] |

*Supplementary Table S2: Due to a lack of crown metrics in ForClim, tree species occurring in ForClim were assigned to a tree species with species-specific coefficients available for crown allometries [1].*

| Tree species with crown allometry available [1] | Assigned tree species occurring in ForClim |
| --- | --- |
| *Picea abies* | ***Picea abies***, *Larix decidua* |
| *Abies alba* | ***Abies alba***, *Taxus baccata* |
| *Pinus sylvestris* | ***Pinus sylvestris***, *Pinus cembra*, *Pinus nigra*, *Pinus montana* |
| *Fagus sylvatica* | ***Fagus sylvatica***, *Acer campestre*, *Acer platanus*, *Acer pseudoplatanus*, *Alnus glutinosa*, *Alnus incana*, *Alnus* *viridis*, *Betula pendula*, *Carpinus betulus*, *Populus tremula*, *Corylus avellana*, *Salix alba*, *Sorbus aria*, *Sorbus aucuparia*, *Tilia cordata*, *Tilia platyphyllos*, *Ulmus glabra* |
| *Quercus petraea* | ***Quercus petraea***, *Quercus pubescens*, *Quercus robur*, *Castanea sativa*, *Fraxinus excelsior* |


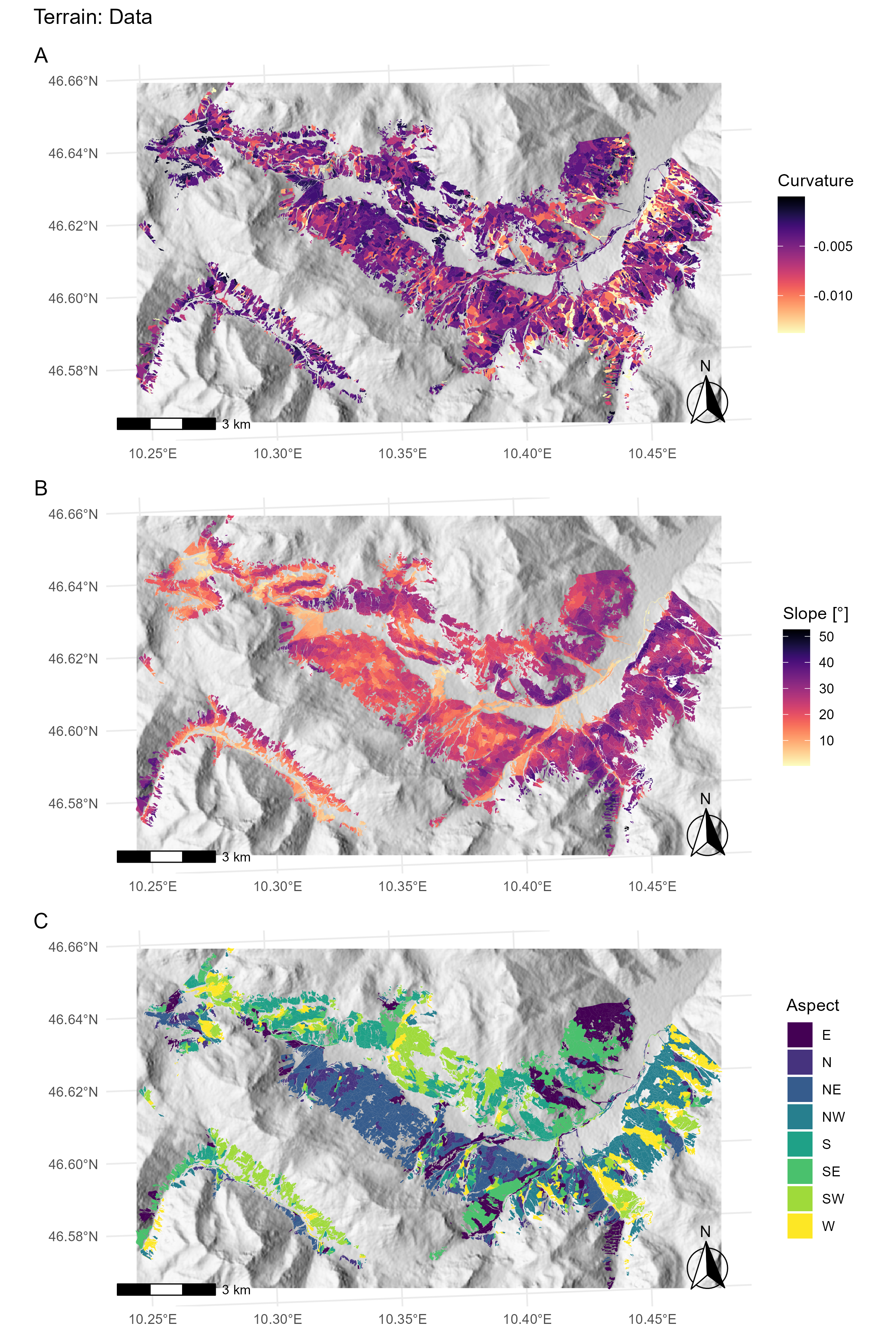


*Supplementary Figure S1: (A) Planar curvature, (B) slope, and (C) aspect for all 5,786 individual forest stands in Val Müstair. Values for slope and aspect were extracted at stand centroid positions.*


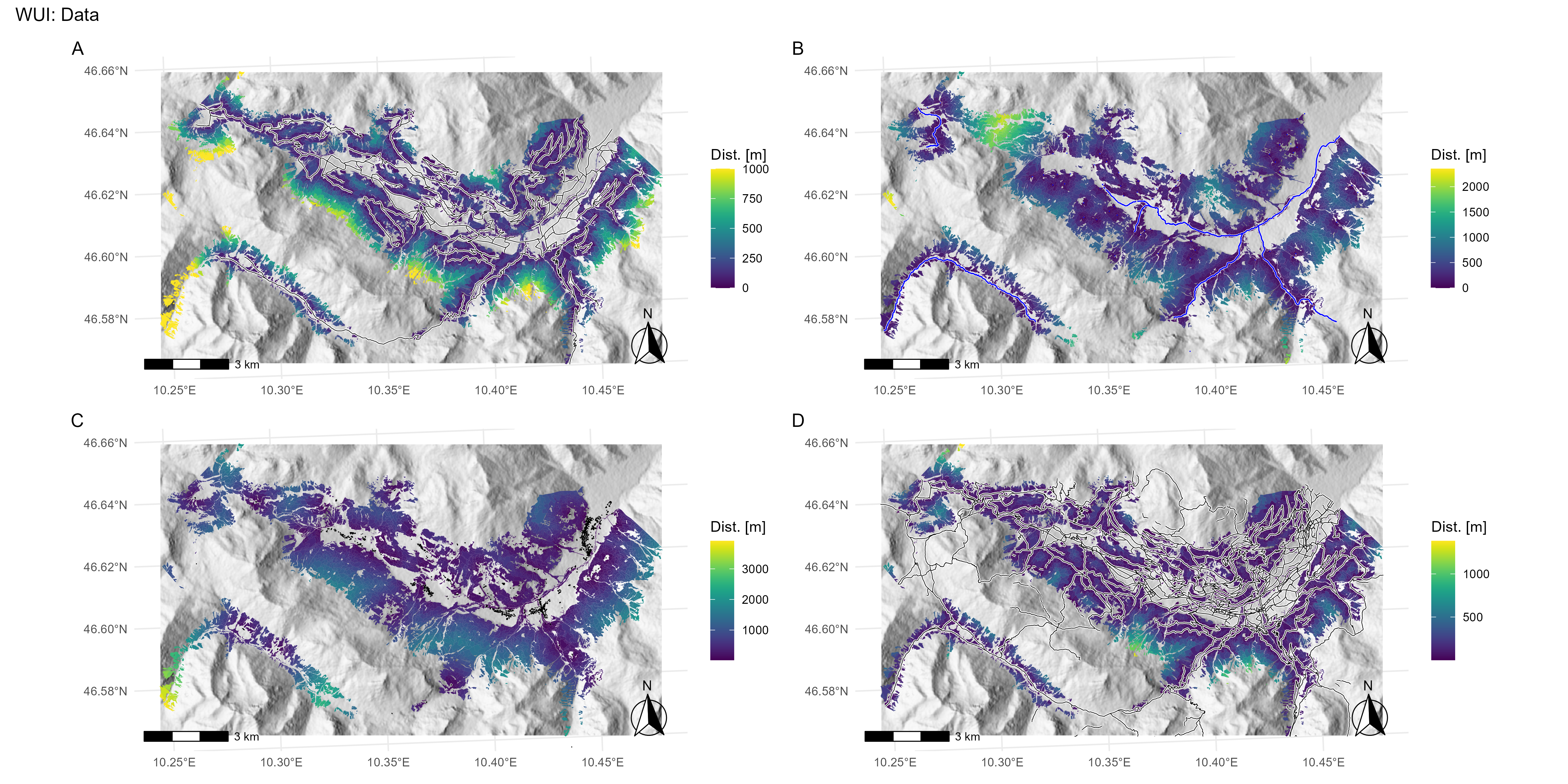


*Supplementary Figure S2: Individual indicators for the assessment of disturbance predisposition arising from the wildland–urban interface (WUI). (A) Distances from forest stands to drivable roads (black lines). (B) Distances from forest stands to the closest waterbody (rivers: blue lines, water extraction points: blue points). (C) Distances from forest stands to the closest building (black dots). (D) Distances from forest stands to the closest road, path, etc. (black lines).*


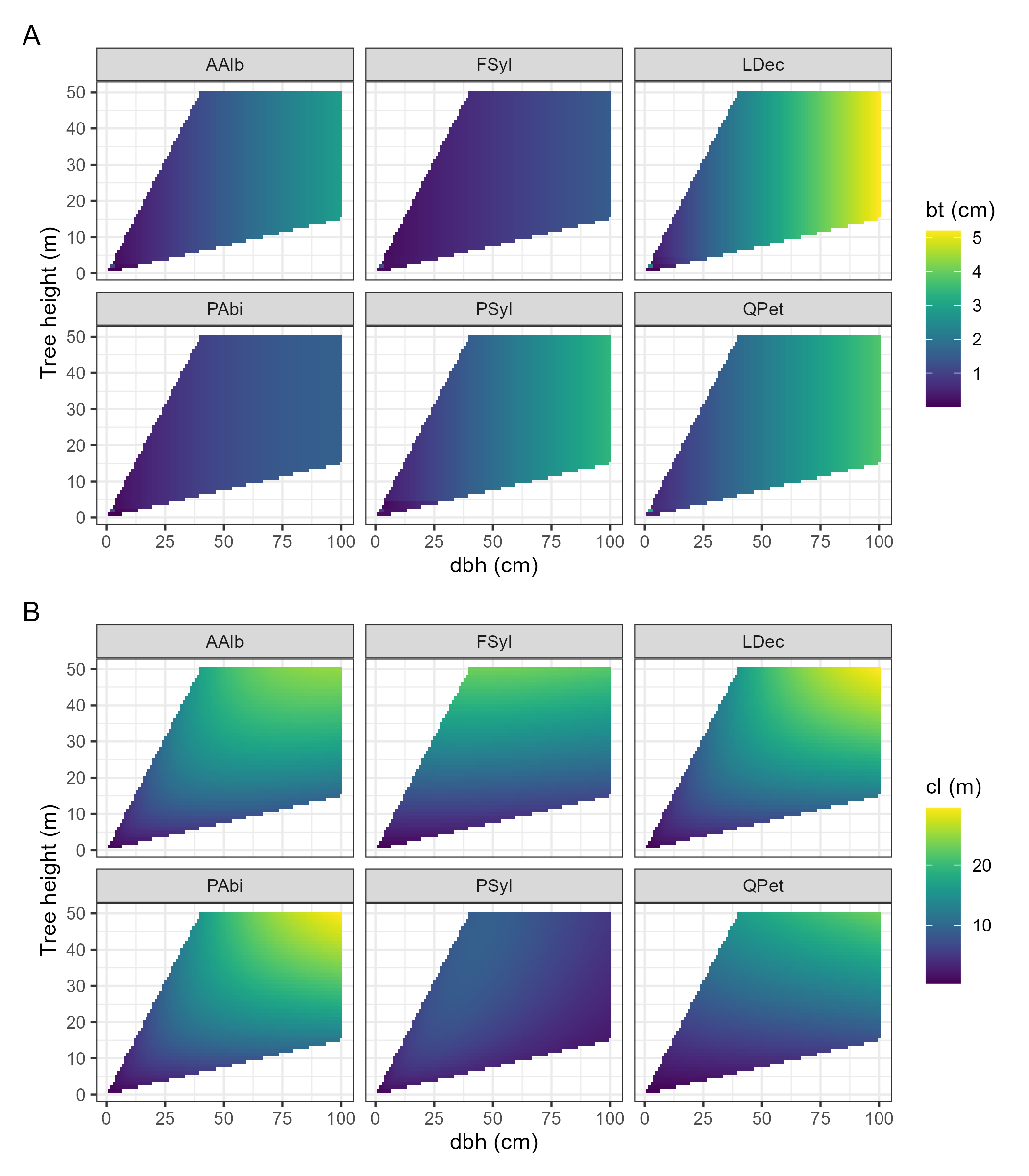


*Supplementary Figure S3: (A) Relationship between tree height, diameter at breast height (dbh), and bark thickness (bt), calculated based on allometries provided in the R package TapeS [2]. (B) Relationship between tree height, diameter at breast height (dbh), and crown length (cl), calculated based on crown allometries [1]. The adopted allometries are shown for six exemplary tree species (Abies alba, AAlb; Fagus sylvatica, FSyl; Larix decidua, LDec; Picea abies, PAbi; Pinus sylvestris, PSyl; Quercus petraea, QPet). For both (A) and (B), values were calculated for systematic combinations of dbh (in steps of 1 cm) and tree height (in steps of 1 m). The obtained values are only shown for tree height to dbh ratios ranging from 0.15 to 1.25.*


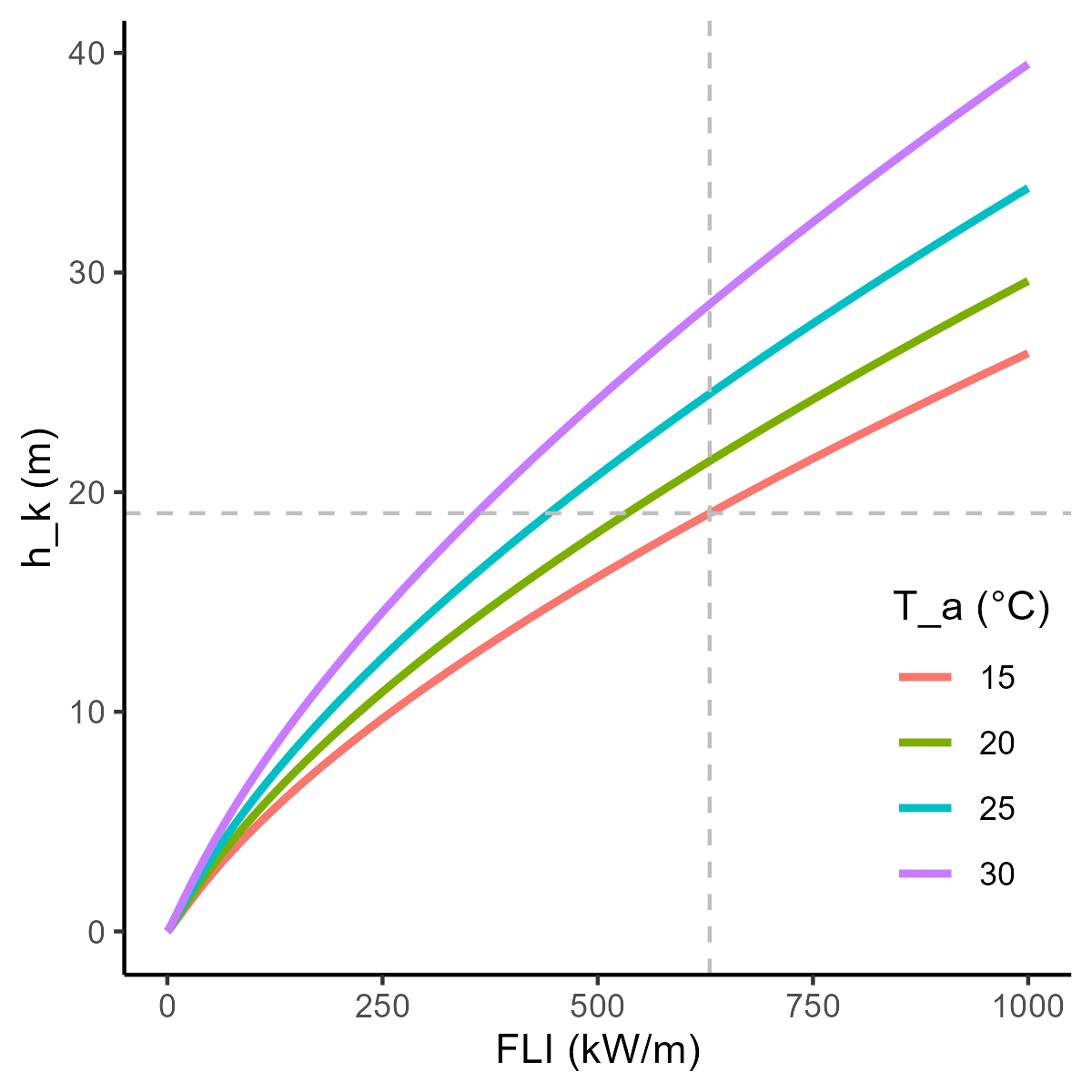


*Supplementary Figure S4: Height of crown scorch (h_k_) as a function of fireline intensity (FLI) for different values of ambient temperature (T_a_)*, *following* *[3]. Assumptions on mid-flame wind speed (MFWS) are based on a default value obtained from the literature [4,5]. The dashed lines represent the default value for h_k_ , used as the basis for further analysis (MFWS: 1.8 m/s, FLI: 630 kW/m, T_a_: 15°C).*

**
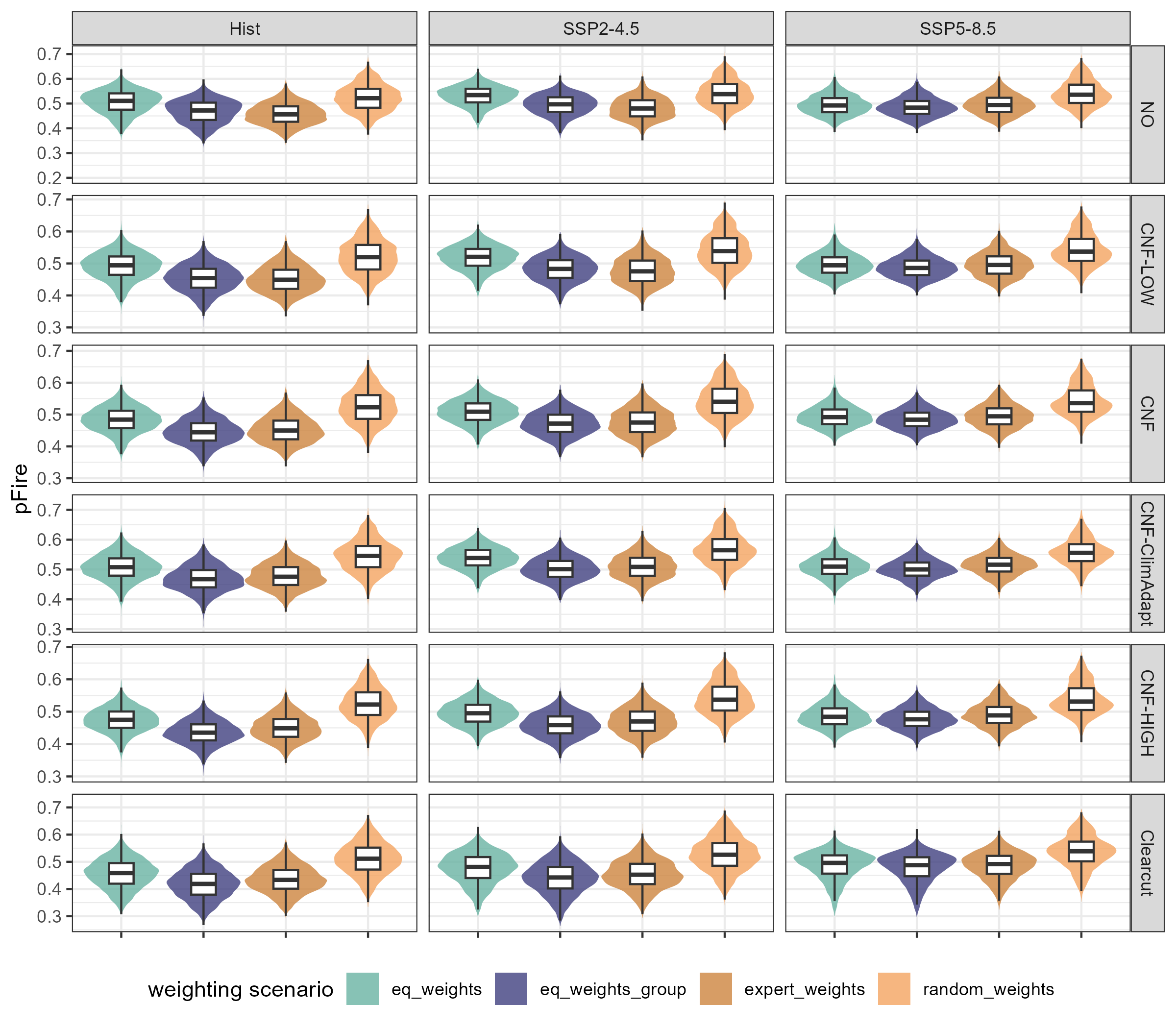
**

*Supplementary Figure S5: Distribution of predisposition to fire (pFire) scores across four weighting scenarios, faceted by simulated silvicultural management (NO, CNF-LOW, CNF, CNF-ClimAdapt, CNF-HIGH, Clearcut) and climate (historical, SSP2-4.5, SSP2-8.5) trajectories. Weighting scenarios: equal weighting of all components (eq_weights), equal weighting at the level of sub-groups (eq_weights_group), expert-defined weights (expert_weights), and randomly assigned weights (random_weights). Violin plots (coloured areas) show the density of pFire, while the overlaid boxplots summarize the median (bold line) and 25th and 75th quantiles (box bounds) of pFire at the end of the simulation period (n=5,786 forest stands, year 2100).*

References

[1] H. Pretzsch, P. Biber, J. Ďurský, The single tree-based stand simulator SILVA: construction, application and evaluation, Forest Ecology and Management 162 (2002) 3–21. https://doi.org/10.1016/S0378-1127(02)00047-6.

[2] C. Vonderach, E. Kublin, G. Kändler, Package ‘TapeS’: Tree Taper Curves and Sorting Based on 'TapeR', 2023.

[3] D.L. Peterson, K.C. Ryan, Modeling postfire conifer mortality for long-range planning, Environmental Management 10 (1986) 797–808. https://doi.org/10.1007/BF01867732.

[4] K. Ryan, Dynamic interactions between forest structure and fire behavior in boreal ecosystems, Silva Fenn. 36 (2002). https://doi.org/10.14214/sf.548.

[5] D.V. Sandberg, C.L. Riccardi, M.D. Schaaf, Fire potential rating for wildland fuelbeds using the Fuel Characteristic Classification SystemThis article is one of a selection of papers published in the Special Forum on the Fuel Characteristic Classification System, Can. J. For. Res. 37 (2007) 2456–2463. https://doi.org/10.1139/X07-093.
